# Geographically weighted machine learning model for untangling spatial heterogeneity of type 2 diabetes mellitus (T2D) prevalence in the USA

**DOI:** 10.1038/s41598-021-85381-5

**Published:** 2021-03-26

**Authors:** Sarah Quiñones, Aditya Goyal, Zia U. Ahmed

**Affiliations:** 1 University at Buffalo, State University at New York, Buffalo, USA; 2 Research and Education in Energy, Environment, and Water (RENEW) Institute, University at Buffalo, State University at New York, 108 Cooke Hall, Buffalo, NY 14260 USA

**Keywords:** Quality of life, Machine learning

## Abstract

Type 2 diabetes mellitus (T2D) prevalence in the United States varies substantially across spatial and temporal scales, attributable to variations of socioeconomic and lifestyle risk factors. Understanding these variations in risk factors contributions to T2D would be of great benefit to intervention and treatment approaches to reduce or prevent T2D. Geographically-weighted random forest (GW-RF), a tree-based non-parametric machine learning model, may help explore and visualize the relationships between T2D and risk factors at the county-level. GW-RF outputs are compared to global (RF and OLS) and local (GW-OLS) models between the years of 2013–2017 using low education, poverty, obesity, physical inactivity, access to exercise, and food environment as inputs. Our results indicate that a non-parametric GW-RF model shows a high potential for explaining spatial heterogeneity of, and predicting, T2D prevalence over traditional local and global models when inputting six major risk factors. Some of these predictions, however, are marginal. These findings of spatial heterogeneity using GW-RF demonstrate the need to consider local factors in prevention approaches. Spatial analysis of T2D and associated risk factor prevalence offers useful information for targeting the geographic area for prevention and disease interventions.

## Introduction

Type 2 diabetes mellitus (T2D), a common and potentially preventable disease, has become a public health concern and imposes significant health and economic burdens in the United States^1^. An estimated 34.1 million adult Americans had T2D in 2018, approximately 13% of the population^2^. T2D is a leading cause of death in the United States with a crude death rate of 24.7 per 100,000 persons^3^. National T2D prevalence is predicted to rise to approximately 14% and 18% in 2030 and 2060, respectively^4^. T2D prevalence varies substantially between and within states in the US, ranging from 2.2% (1.3–3.2%) to 28.7% (15.8–44.2%) in 2017^5^. State- and county-level variations in T2D prevalence reveal many shortcomings in individual-level considerations of diabetes risk factors and intervention strategies. The spatial modeling of the association between T2D prevalence and its risk factors and establishing how this association varies over space is important for identifying geographical areas that would benefit from specific efforts and resources to reduce the T2D burden.

A number of spatial modeling studies have demonstrated associations between county-level T2D prevalence and several socioeconomic and lifestyle factors such as poverty^1,6,7^, obesity^8–10^, physical inactivity^1,9^, and food environment^11–13^. Spatial analysis of T2D prevalence and many of these risk factors offers useful information in health care promotion programs and public policy decisions^14,15^. Geographically weighted ordinary least squares regression (GW-OLS), an extension of linear regression^16,17^, has been widely used to explore geographic variations in risk factors and diabetes prevalence^10,18–20^. However, GW-OLS is not appropriate to estimate the relationships between predictors and target variables when their relationship is non-linear, and local multicollinearity among the predictors exists^21^, as is the case with T2D. The relationship between risk factors and T2D prevalence is complicated and not always linear^22^. It is necessary to deal with the nonlinear situation in a local regression model to explore the spatial variation of T2D prevalence in relation to risk factors.

The geographically weighted random forest (GW-RF), a tree-based non-parametric ensemble model, has been recently developed to address the limitations of the GW-OLS model and improve predictive performance over a non-geographically weighted random forest (RF) model^23^. The main idea of GW-RF is similar to that of the GW-OLS model^17^, in which the model is calibrated locally rather than globally. The GW-RF does not need to consider multicollinearity and can analyze all independent variables without screening^24^ and may provide superior predictive power and evaluation of associations between independent and dependent spatial variables compared to the GW-OLS^23^. However, applying a non-parametric GW-RF model to explain spatial heterogeneity between disease outcome and risk factors is still lacking, and further research comparing GW-OLS and GW-RF is therefore warranted.

To our knowledge, no studies have assessed the advantages of GW-RF to explore non-stationarity in the relationships between county-level T2D prevalence and risk factors. This study applied the GW-RF model to explore and visualize non-stationarity in the relationship between T2D and selected risk factors. Our objectives were (i) to explore the local associations between T2D prevalence and risk factors for targeting the geographic area for prevention and interventions and (ii) to evaluate the predictive performance of GW-RF compared to traditional local and global models.

## Materials and methods

### Data

County-level age-adjusted adult (> 18 years) T2D, obesity, and physical inactivity prevalence from years 2013 to 2017 were obtained from United States Diabetes Surveillance System (USDSS)^5^. Data from the CDC's Behavioral Risk Factor Surveillance System (BRFSS) and the US Census Bureau's Population Estimates Program were used to estimate the county-level prevalence of diabetes, obesity, and physical inactivity^5,25^. The BRFSS is a national, monthly administered telephone survey that collects data at the state-level on disease risk factors and preventative health behaviors. The BRFSS utilizes separate sampling procedures for landline telephone respondents and for cellular telephone respondents^5^. Disproportionate stratified sampling is used for the landline sample for all years of data in this analysis. Telephone numbers are drawn from two strata of telephone number density: (1) high density or (2) medium density. The landline sampling ratio of high to low-density residential numbers in the BRFSS is 1:1.5. Disproportionate stratified sampling is more efficient than simple random sampling. The cellular telephone sample is selected randomly from a sampling frame of confirmed area codes and prefix combinations. Cellular respondents are randomly selected with an equal probability of being selected into the sample. Landline telephone numbers are sampled by the BRFSS based on sub-state geographic regions to account for small-area differences within states. The BRFSS two-step weighting process of design weighting followed by iterative proportional fitting is undertaken to remove bias. Summary data quality reports released with the BRFSS data each year show median response rates between 40.5 and 48.7% between 2014 and 2016 for landline and cellular telephone responses. A positive answer to the question determined diabetes prevalence in this survey, "Has a doctor ever told you that you have diabetes?" Women who reported diabetes during pregnancy were omitted. Age-adjusted percentages of diagnosed diabetes among adults 18 years or older are presented at the county level. Bayesian multilevel modeling was used to estimate diabetes, obesity, and physical inactivity prevalence at the county-level^26,27^. There is an expectation of 5% disagreement in the model due to sampling variability. Estimates of county-level prevalence were age-adjusted using the 2000 United States standard population using the following age groups: 20–44, 45–64, and 65 and older^28^. Since T2D accounts for 90–95% of all types of diabetes, we have used T2M to represent USDSS county-level diabetes prevalence.

In the BRFSS survey, respondents were also asked, "During the past month, other than your regular job, did you participate in any physical activities or exercises such as running, calisthenics, golf, gardening, or walking for exercise?" If the response was "no," respondents were considered physically inactive. Obesity was determined by a body mass index of 30 kg/m^2^ or higher, which was calculated using the self-reported height and weight of the participants. The prevalence of both obesity and physical inactivity was defined as the age-adjusted percentage of adults 20 years or older that were considered obese or physically inactive in a given county.

County-level, age-adjusted poverty data (% population below poverty level) were obtained from the US Census Bureau's Small Area Income and Poverty Estimates (SAIPE) program, which provides annual estimates of income and poverty for all school districts, counties, and states. A regression model was used to predict the number of people in poverty using single-year county-level observations from the American Community Survey (ACS) as the dependent variable, and administrative records and census data as the predictors^29^.

The percentage of the county population who have some post-secondary education was obtained from County Health Ranking^30^. The County Health Rankings use 5-year averages of the ACS data to get measures of social and economic factors. ACS is an ongoing survey program of the Census Bureau that provides vital information about population and housing information about the country. These percentages were subtracted from 100 to calculate the percentage of individuals in a given county with no higher education—our measure of poor education.

Food environment index data were retrieved by County Health Rankings from the United States Department of Agriculture (USDA) Food Environment Atlas and the Map the Meal Gap from Feeding America for the years 2013–2017^31,32^. The Food Environment Index ranges from 0 (worst) to 10 (best) and equally weighs two indicators of the food environment: limited access to healthy foods and food insecurity. These two indicators provide county-level healthy food access and availability measures based on store/restaurant proximity, food prices, food and nutrition assistance programs, and community characteristics^33^.

Access to exercise opportunities is a measure of the percentage of individuals in a county living within reasonable proximity to a location conducive for physical activity^34^. Individuals are considered to have access to exercise opportunities if they: reside in a census block that is within a half-mile of a park, reside in an urban census block that is within one mile of a recreational facility, or reside in a rural census block that is within three miles of a recreational facility. Five years (2012–2016) of data of the percentage of the population with access to exercise were obtained from County Health Rankings^33^. County Health Rankings use the North American Information Classification System (NAICS) which was then combined with Business Analyst data in ArcGIS^35^, Delorme map data, ESRI, and US Census Tigerline files.

### Exploratory data analysis

#### Hot spot analysis (Getis-Ord Gi*)

We calculated the Getis-Ord Gi* statistic to identify hot spots (high values) and cold spots (low values) for diabetes in ArcGIS Spatial Statistics Tools^35^. The estimated *z*-scores and *p*-values calculated in each county indicate areas with either high or low-value clusters. Larger z-scores (statistically significant positive z-scores) show more intense clustering of high values, and smaller z-scores (statistically significant negative *z*-scores) indicate more intense clustering of low values.

#### Bivariate Local Moran I

To explore the degree of linear association (positive or negative) between T2D prevalence and risk factors at a given location and the average of another variable at neighboring areas (spatial lag), we estimated bivariate Local Moran I (LMI) statistics, which provide a classification of four types of spatial autocorrelation. LMI does not control the correlation between variables at each location, but instead identifies counties with significant clusters (at α = 0.05) for T2D prevalence and any risk factors at the same time^36^.

#### Geographically weighted correlation

To investigate local relationships between T2D prevalence and risk factors, we calculated geographically weighted (GW) Pearson's correlation coefficients using the methods described by Brunsdon^16^. The GW correlation coefficients provide a preliminary assessment of non-stationarity relationship between the dependent and an independent variable of a GW regression^37^. We used "bi-square kernels" with adaptive distance to select the optimum neighbor size. The "GWModel" package^38^ in the R statistical computing environment^39^ was used for this analysis.

### Geographically weighted model

#### Geographically weighted OLS regression (GW-OLS)

The GW-OLS involves spatial regression techniques increasingly used when data are not described well by a global model^17^. GW-OLS explores spatial heterogeneity in the relationships between variables where non-stationarity exists such that locally weighted regression coefficients move away from their global values. GW-OLS fits a regression equation for every location in the dataset, incorporating the dependent and explanatory variables falling within the user-selected bandwidth of each target location. The bandwidth's shape and size usually depend on the kernel type, bandwidth method, distance, and the number of neighbors parameters. Like GW correlation analysis, we used "bi-square kernels" with adaptive distance to select the optimum neighbor size. We found the lowest AICc values at 248 nearest neighbors county (Figure S3). For evidence of local coefficient estimates significantly different from zero, we calculated *p*-values (adjusted) from pseudo-*t*-values using the method described by Fotheringham-Byrne^40^. To investigate local collinearity in a GW regression model, we also calculated local variance inflation factors (VIFs) for each independent variable. Local collinearity problems in the GW regression model are usually considered if VIFs greater than ten would be found at any locations for any independent variables^37^. The "GWModel" package^38^ in R Statistical Computing Environment^39^ was used for this purpose.

#### Geographically weighted random forest (GW-RF)

The linear model is susceptible to outliers, and strong assumptions are required regarding the relationships between predictors and target variables (linearity) and the relationships among the predictors (collinearity). The nonlinear non-parametric models such as random forest (RF) do not need to consider multicollinearity and can analyze all independent variables without screening^24^. The geographically-weighted random forest (GW-RF) model may address the limitations of the linear GW-OLS model and can improve predictive performance relative to a non-geographically-weighted random forest model, which is unable to resolve heterogeneous spatial processes^23^. The main idea of GW-RF is similar to that of the traditional GW-OLS model, in which the model is calibrated locally rather than globally^17^ by integrating spatial weight matrix (SWM) and RF into a local regression analysis framework^24^. The local feature importance represents the mean increase in Mean Squared Error (incMSE) if a predictor would be randomly permuted or the decrease in node impurities (IncNodePurity) from splitting on the variable, averaged over all trees. Both measures are derived from the Out of Bag (OOB) error. More details on these model approaches can be found in the supplemental materials.

Before fitting the GW-RF model, we used a Random Grid Search (RGS) to find the optimal *parameters* for the global RF model. We employed the *K*-fold cross-validation method to determine the optimal hyper-parameters from a set of all possible hyper-parameter value combinations (Supplementary Information Table S2). During the parameter tuning process for the early stopping parameters, we used 0.001 and 2 for "stopping tolerance" and "stopping rounds", respectively. The best parameters ("ntree", number of tree and "mtry", number of variables randomly sampled) for global RF model were used to train the local GW-RF model. We trained the GW-RF with 284 nearest neighbors with bootstrapped 2950 "ntrees" and 4 "mtry" in each tree. Both the global and local RF models were trained with mean data from 2013 to 2017, 2013–2015, and of 2016 and 2017 to explore variation in feature importance due the data distribution. We used the two most commonly used global interpretability approaches, such as the Permutation Feature Importance (PFI) approach^41^ and partial dependency profile^42^, to interpret the predictors' role in the global RF model. We also ranked the variables based on the mean decrease Gini impurity index or “IncNodePurity” that is used for the calculating the splits in trees. We used "SpatialML" package^23,43^ in the R Statistical Computing Environment^39^.

for GW-RF analysis. For feature importance and generated partial dependence profiles (PDP) global RF model, we used the "DALEX" package^44^ in R Statistical Computing Environment^39^.

#### Predictive performance of GW models

Like other regression models, GW-RF can also be used as a predictive model rather than a tool to explore spatial heterogeneity in the relationship between disease outcomes and risk factors. We first evaluated the predictive performance of GW-RF using K-fold cross-validation. Cross-validation statistics usually give a better indication of how a model will perform on unseen data. In *K*-fold cross-validation, the data set was randomly divided into a test and training set *k* different times, and model evolution was repeated *k* times. Each time, one of the *k* subsets was used as the test set, and the other *k-*1 subsets are put together to form a training set. Then the average error across all *k* trials was computed. Diagnostic measures of *K*-fold cross-validation were root-mean-square error (RMSE) and goodness of fit (R^2^). We also evaluated GW-OLS and GW-RF performance to predict county-level T2D prevalence using a sub-set of data. The data set (n = 3108) was randomly split into 2484 training data used for training again the GW models and 624 test data (Figure S2), which were used to evaluate the models. The summary statistics and distribution of T2D prevalence and risk factors of training and test data sets are reasonably close to the entire data set (supplementary data, Table S1 and Figure S2). Global Ordinary least squares (OLS) and RF regression models were used as benchmark methods.

## Results

### Exploratory data analysis

**Figure 1:**
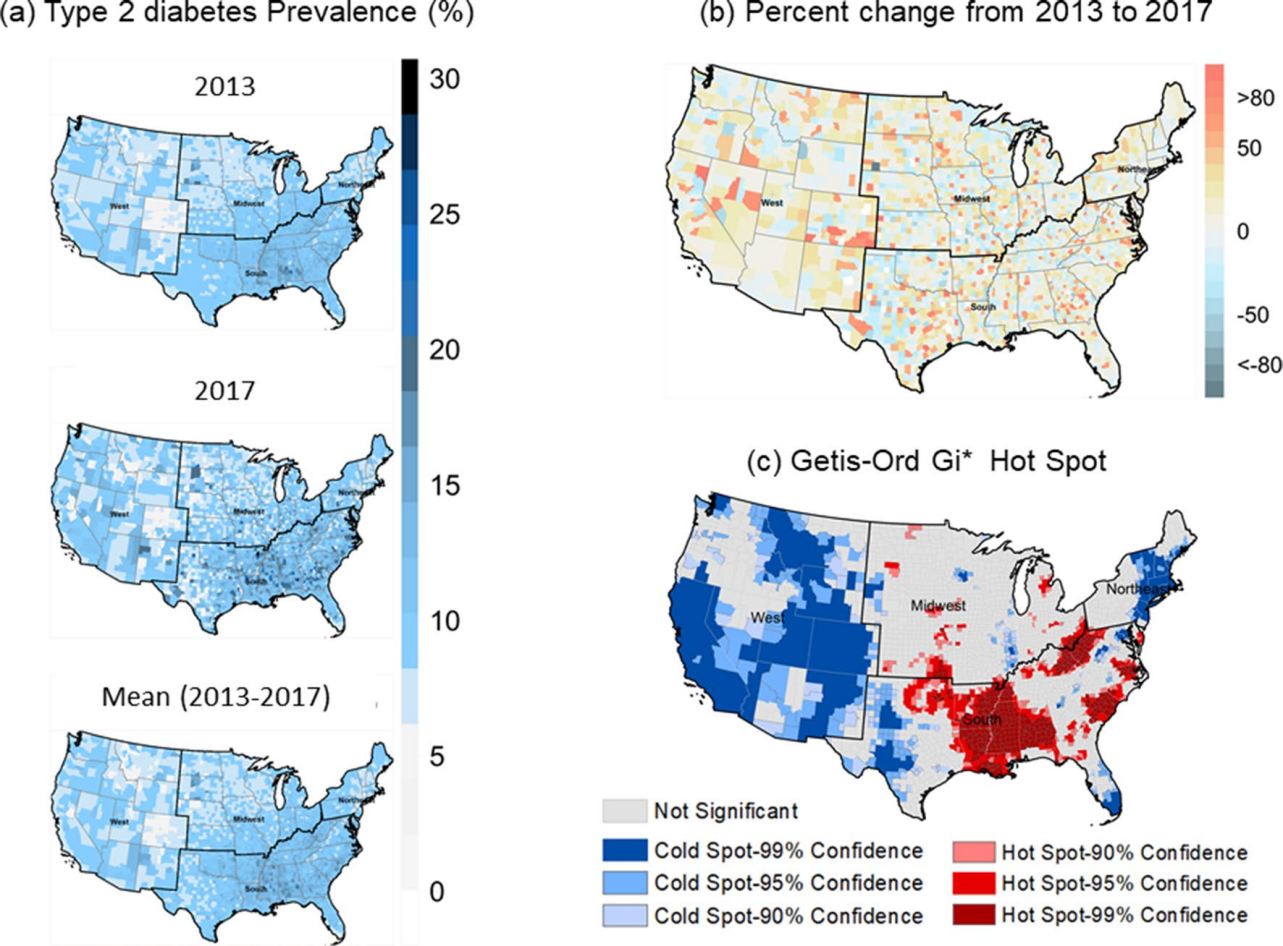
County-level prevalence maps of (**a**) T2D for the years 2013–2017 and 5-year average (2013–2017); (**b**) percent change from years 2013–2017; and (**c**) the geographical clusters of counties from Getis-Ord Gi* statistics of T2D. Maps in (**a**) and (**b**) were created in the R (4.0.0) Statistical Computing Environment^39^. Getis-Ord Gi* Hot Spot map was created in ArcGIS Desktop version 10.6.1^35^.

Figure 1a shows the spatial distribution of county-level, age-adjusted T2D prevalence in the years 2013 and 2017, and 5 year mean (2013–2017) (Fig. 1a). County-level prevalence of T2D remains relatively stable through these years, with notable increases in the year 2017 concentrated in the southeast US. Averages of T2D prevalence at the county-level over the years 2013–2017 show higher prevalence (> 10%) in many counties of the southeast US while many western counties tend to haveT2D prevalence below 10%. Changes in T2D prevalence between 2013 and 2017 were mostly positive in many counties scattered throughout the US, experiencing more than a 50% increase in prevalence over the 5 years, while few counties show declines T2D (Fig. 1b). Getis-Ord Gi* Hot Spot analysis shows high clustering of T2D in the southeast US while there are cold (low) clusters of T2D in the West and regions of the Northeast (Fig. 1c).

**Figure 2:**
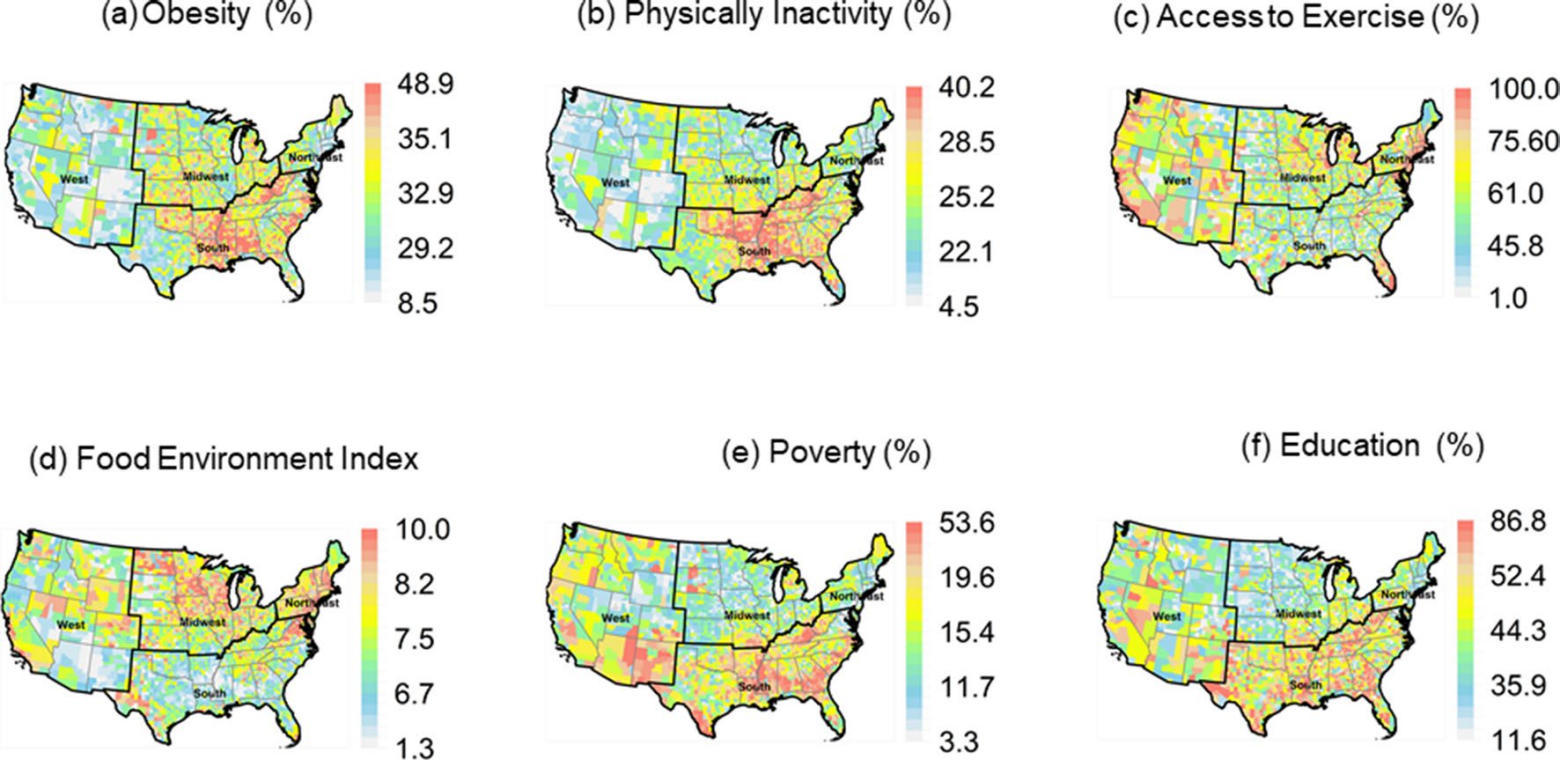
County-level 5-year averages (2013–2017) of six risk factors. (**a**) obesity; (**b**) physical inactivity; (**c**) access to exercise; (**d**) food environment index; (**e**) poverty; and (**f**) education. Maps were created in the R (version 4.0.0) Statistical Computing Environment^39^.

The mean values of six risk factors of T2D risk from 2013 to 2017 are shown in Fig. 2. Prevalence of obesity, physical inactivity, and lack of higher education generally share similar geographical locations; the highest being in the southeast US counties (Fig. 2a,b,f). In contrast, counties in the West, Midwest, and Northeast tend to have lower mean obesity and physical inactivity, and better educational attainment. Percent of people who have access to exercise (Fig. 2c) is generally high across the US counties in the West and Northeast regions (75–100%). Poverty also seems to be ubiquitous across US counties, though there are apparent clusters of high poverty in the South and West (Fig. 2e). Food environment index across the US indicates that populations in the Midwest and Northeast have the better access to food outlets and healthy foods compared to the West and Southeast (Fig. 2d).

**Figure 3:**
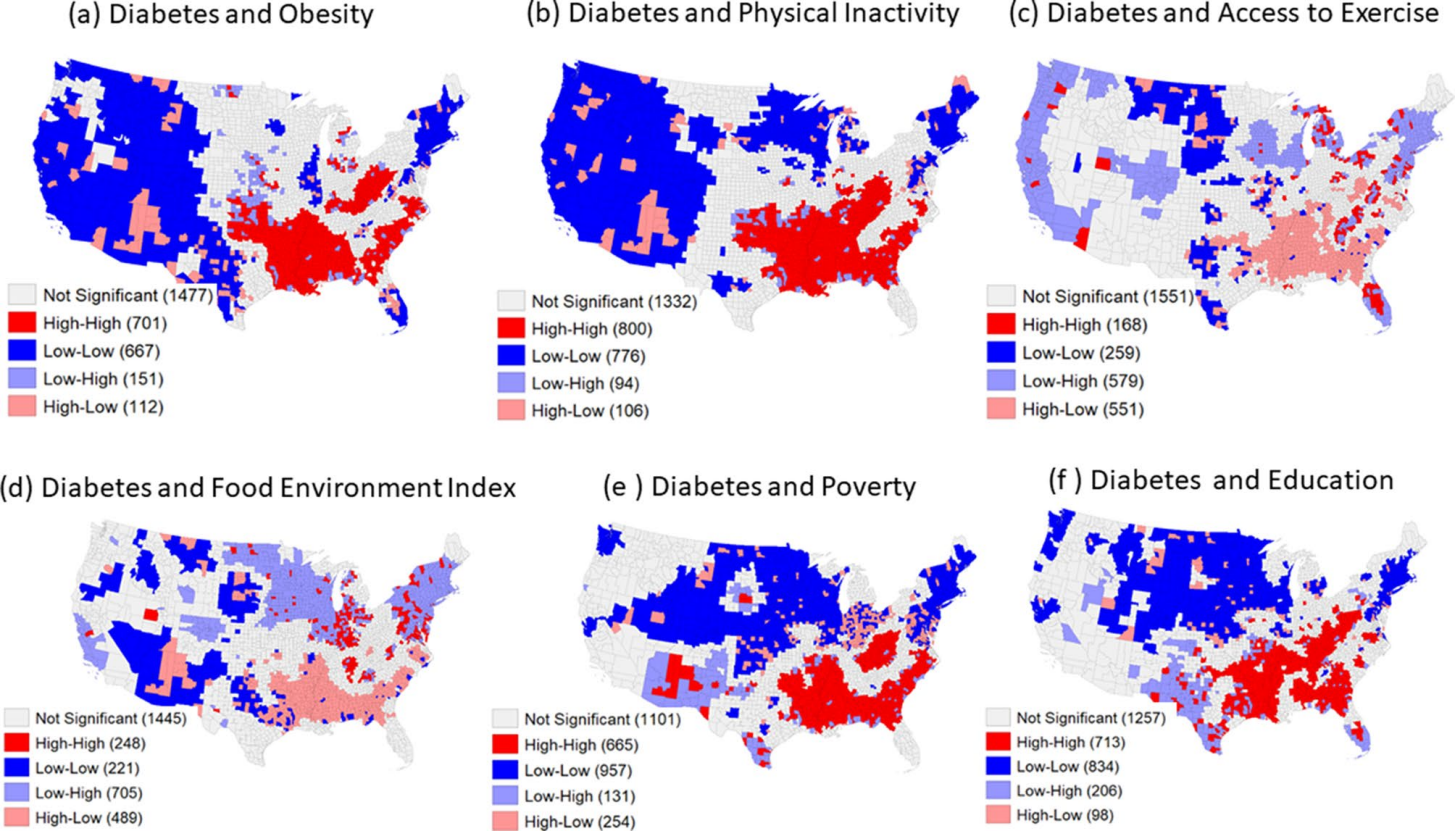
Bivariate LMI cluster of diabetes and (**a**) obesity; (**b**) physical inactivity; (**c**) access to exercise; (**d**) food environment index; (**e**) poverty; and (**f**) education. Maps were generated in GeoDa (version 1.14), an open source software for geodata analysis^45^.

The bivariate global Moran's I result in Figure S4 show a positive association between T2D and physical activity, obesity, poverty, poor education, and a negative association between access to exercise and the food environment index. LMI clusters of T2D and these 6 risk factors are presented in maps in Fig. 3. The red color (High-High) correspond to significant clusters of high T2D prevalence with a high prevalence of obesity (Fig. 3a), physical inactivity (Fig. 3b), poverty (Fig. 3e), and education (Fig. 3f). These counties are mostly concentrated in the South. The light red color (High-Low) in maps represents significant clusters of high DM-2 prevalence with limited access to exercise (Fig. 3c) and low food environment index (Fig. 3d).

**Figure 4:**
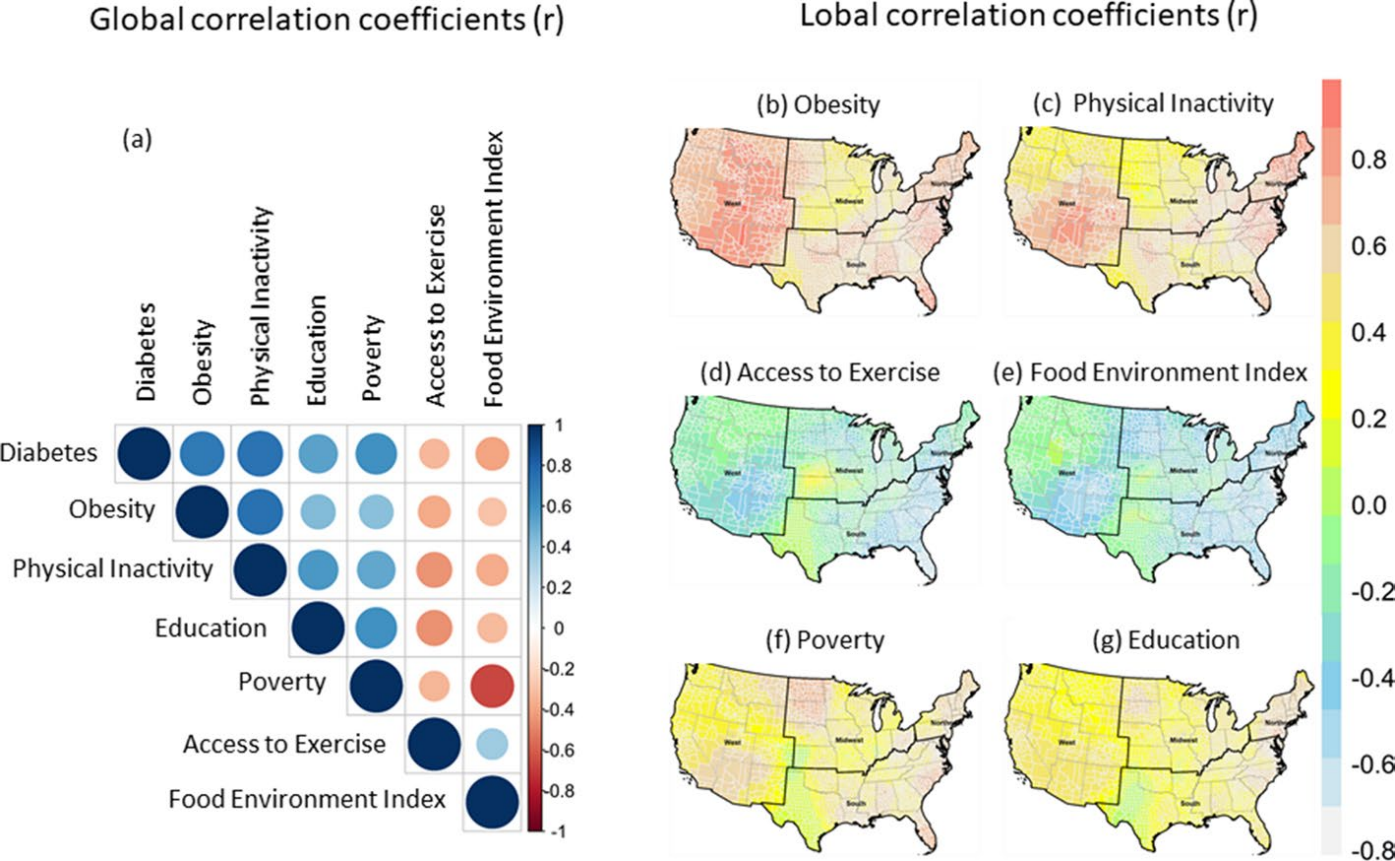
Global (**a**) and local Pearson correlation coefficients (*r*-values) of T2D prevalence and six risk factors. (**b**) obesity; (**c**) physical inactivity; (**d**) access to exercise; (**e**) food environment index; (**f**) poverty; and (**g**) education. Maps were created in the R (version 4.0.0) Statistical Computing Environment^39^.

Local correlation analysis was used to explore the relationship between T2D and the six major risk factors at any county with a specific bandwidth (Fig. 4). The global correlations between T2D prevalence and six risk factors are moderate to strong (Fig. 4a). The correlations were positive for T2D and obesity, physical inactivity, and poverty, and education level and negative for T2D and access to exercise and food environment index. These relationships, however, showed non-stationarity and varied spatially (Fig. 4b–g). For instance, the local correlation between obesity and T2D is strong in counties in the West, but weak in the Midwest (Fig. 4b).

Similarly, physical inactivity has positive and strong correlations with T2D in the West, Northeast, and South (Fig. 4c). Local correlations between T2D and access to exercise and food environment index are generally similar and moderate; both predictors are negatively associated with T2D in the West, South, and Midwest (Fig. 4d,e). Poverty and low educational attainment generally have weak and positive correlations with T2D in most counties (Fig. 4f,g).

### Geographically weighted ordinary least squares (GW-OLS)

All risk factors were positively associated with T2D prevalence (*p* < 0.001) in the global OLS model, except for the food environment index (Table 1). Although the global OLS model explained 67% variability of T2D prevalence, it provided a baseline for subsequent global and local models. Local OLS (GW-OLS) showed significant improvement over the global OLS model, reflected by higher adjusted R^2^ values and lower AIC values (Table 1). The local R^2^ values are relatively high in most counties in the West and some counties in the Midwest, South and Northeast (Figure S11a).

**Table 1:** Summary results of ordinary least square (OLS) and geographically weighted ordinary least squares regression (GW-OLS) models.

	OLS		GW-OLS				
	Estimate	Pr( >|t|)	Min	1st Qu	Median	3rd Qu	Max
Intercept	10.02	< 2e−16***	8.40	9.46	10.02	10.82	11.57
Obesity	0.82	< 2e−16***	0.12	0.67	0.87	1.05	1.65
Physical inactivity	0.85	< 2e−16***	− 0.12	0.28	0.58	0.85	1.22
Access to exercise	0.20	5.36e−11***	− 0.56	− 0.09	0.02	0.16	0.60
Food environment index	− 0.02	0.662^NS^	− 1.02	− 0.29	− 0.14	0.03	0.50
Poverty	0.18	1.66e−06***	− 0.58	− 0.11	0.02	0.16	0.58
Education	0.65	2e−16***	− 0.40	0.14	0.35	0.58	1.21
R^2^		0.665					0.791
Adjusted-R^2^		0.665					0.770
AIC		11,035.6					9978.4

OLS: ordinary least square**s**, GW-OL**S**, geographically weighted OLS regression, AIC: Akaike's information criterion.

***p < 0.001, NS: not significant.

**Figure 5:**
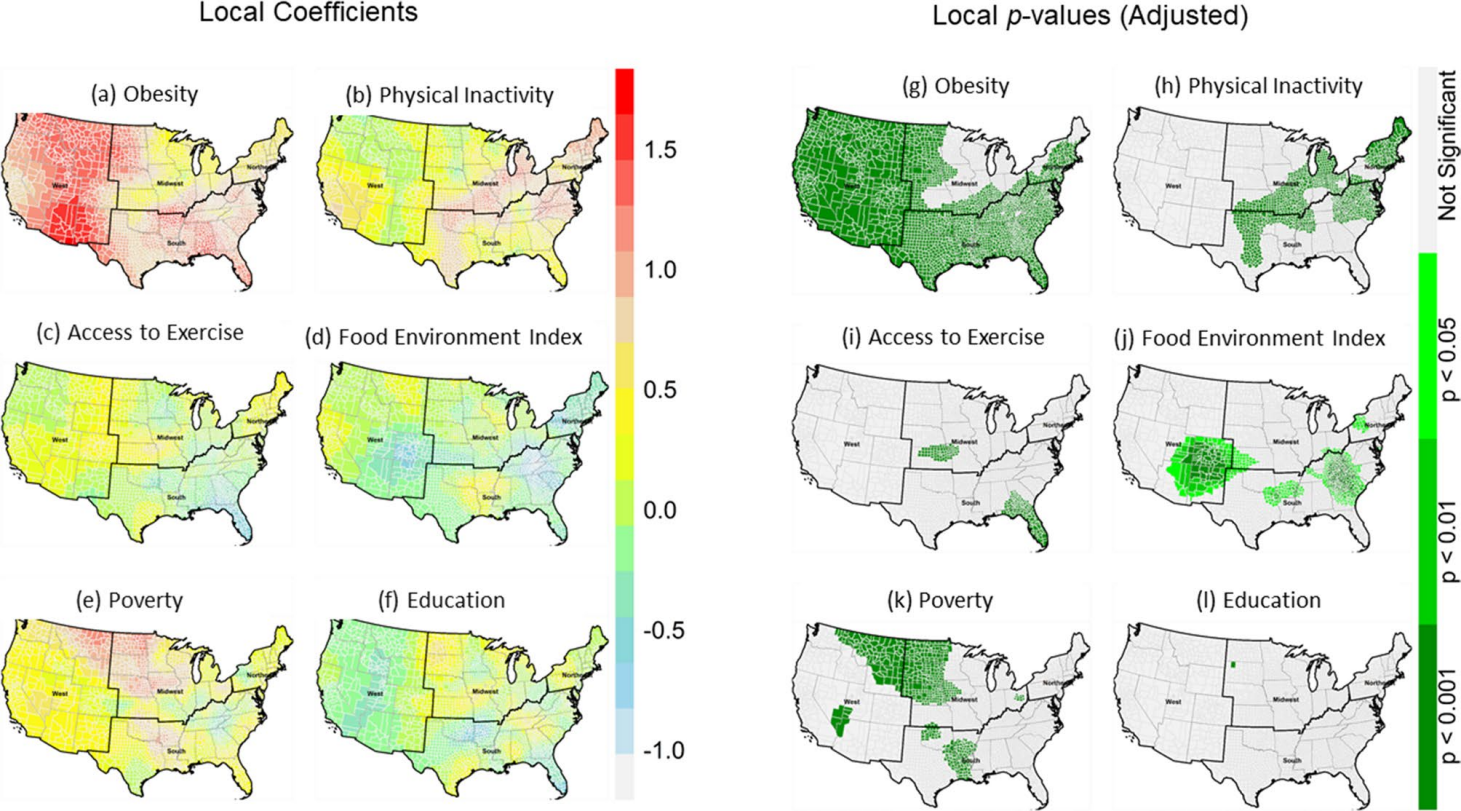
Spatial variation of local coefficients and *p*-values (adjusted) of geographically weighted OLS (GW-OLS) regression models. (**a**–**f**) local coefficients of obesity, physical inactivity, access to exercise, food environment index, poverty and education, and (**g**–**l**) corresponding local *p*-values of all predictors. Maps were created in the R (version 4.0.0) Statistical Computing Environment^39^.

The local GW-OLS VIFs for each independent variable suggests that there is negligible collinearity as no value exceeds 10 for any of the risk factors (Supplementary Figure S5). Local VIF values for obesity and physical inactivity appear slightly higher in the lower Western region than the rest of the country, being the only region with values over 5 for these risk factors. Local coefficients for obesity ranged from 0.12 to 1.65 with a median value of 0.87 (Table 1), and the coefficients are significant and positive in 80% of counties in the conterminous USA (Figure S6a). However, the high values were generally in many counties in the West and South (Fig. 5a,g). Compared to obesity, the coefficients for physical inactivity were narrower, ranged from 0.28 to 1.22, and statistically significant in some counties in the Northeast, Midwest, and South (Fig. 5b,h), which represents 39% of counties in the conterminous USA (Figure S6b). Positive and significant coefficients for poverty were found in the Midwest and West (Fig. 5e,k). A small number of counties showed statistically significant negative coefficients for access to exercise (Fig. 5c,i) and food environment index (Fig. 5d,j). A small number of the counties showed a significant positive coefficient for education (Fig. 5f,l).

### Geographically weighted random forest regression (GW-RF)

The GW-RF model showed lower MSE values than that of global RF (Table 2). The local pseudo-R^2^ ranged from 0.18 to 0.78, with a mean value of 0.50. The local GW-RF models are more robust (pseudo-R^2^ > 0.6) in 28% of counties in parts of the West and Northeast regions (Figure S11b). At the same time, they become less accurate (pseudo-R^2^ < 0.5) in 48% of counties in the central Midwest and South. The result suggests that additional variables should be included to improve the GW-RF model performance in these regions of the US.

**Table 2:** Summary results of random forest (RF) and geographically weighted random forest regression (GW-RF) models.

	RF	GW-RF			
	%IncMSE	Min	Max	Mean	Std
Obesity	175.60	2.33	179.98	79.75	33.94
Physical inactivity	178.85	6.76	115.92	56.95	24.29
Access to exercise	86.85	− 13.01	46.37	14.35	11.68
Food environment index	81.24	− 3.96	55.06	21.94	10.22
Poverty	116.09	− 7.31	64.90	21.85	13.59
Education	143.86	6.34	84.48	37.03	13.09
MSE	1.87	0.75	2.79	1.69	0.48
R^2^	0.69	0.184	0.780	0.508	0.005

Both models were trained with 5 years of mean data (2013–2017) of 3108 counties.

%IncMSE: mean increase in MSE, MSE: mean squared error.

**Figure 6:**
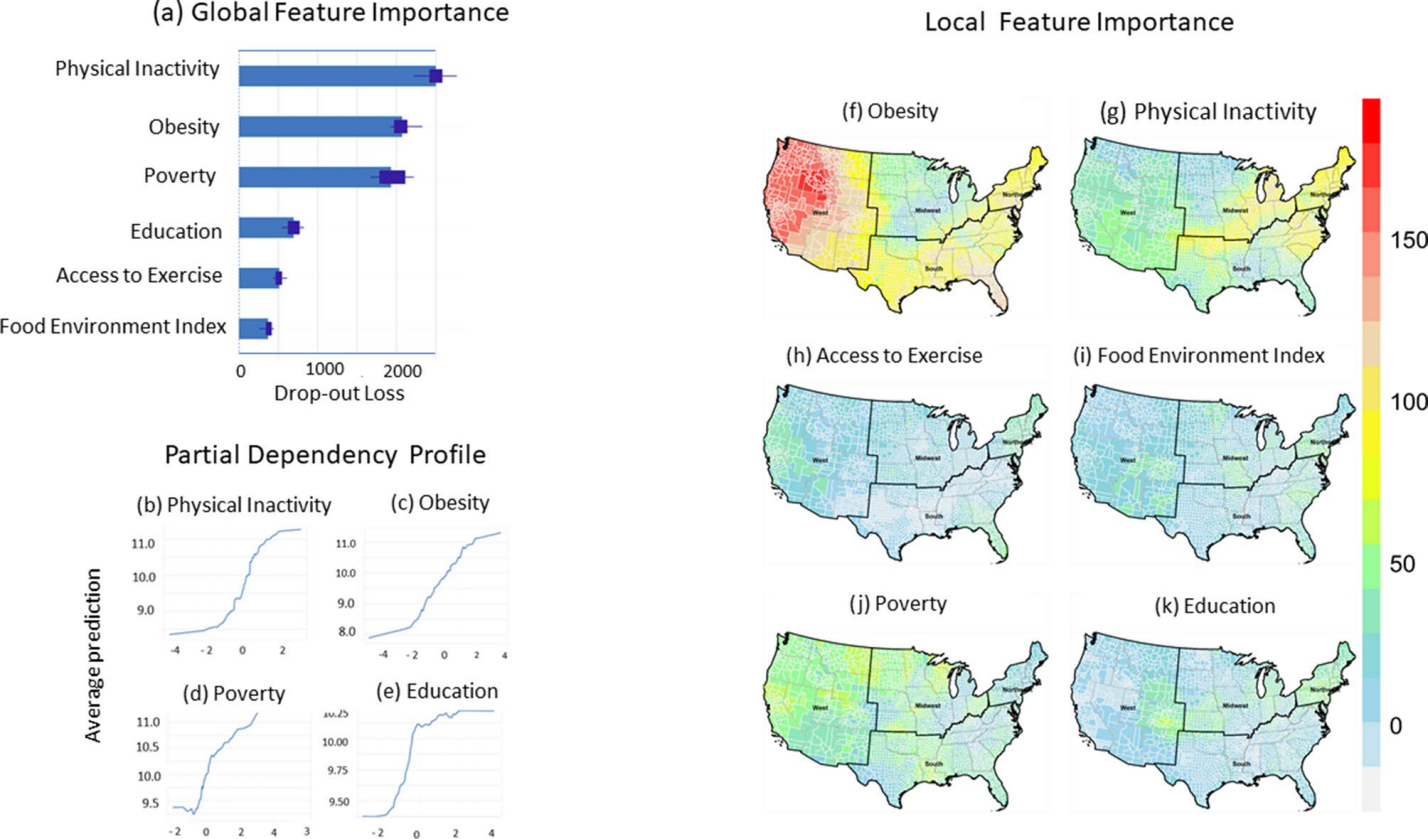
(**a**) Permutation-based feature importance from global random forest, (**b**,**c**) partial dependency profiles of the first four important variables of global random forest model, and (**f**–**l**) spatial variation of local feature importance (%incMSE) of obesity, physical inactivity, access to exercise, food environment index, poverty, and education in geographically weighted random forest regression models. Higher values imply increased importance. The random forest model was trained with 5 years of mean data (2013–2017) of 3108 counties. Maps were created in the R (version 4.0.0) Statistical Computing Environment^39^.

The Permutation-based Feature Importance (PFI) (Fig. 6a and Table 1) and mean decrease Gini score or IncNodePurity (Figure S7a) ranked physical inactivity as the number one most important variable, followed by obesity, poverty, and education. This ranking is consistent for the mean data from 2013 to 2015 (Figure S8a), and data for 2016 (Figure S9a) and 2017 (Figure S10a). When the effect of other predictors was controlled for, the impact of physical inactivity (Fig. 6b), obesity (Fig. 6c), poverty (Fig. 6d), and education (Fig. 6e) on T2D prevalence generally increased throughout their ranges.

The high incMSE values (> 100) for obesity were observed in a large number of counties in the West, South, and Northeast regions (Fig. 6f), which implies increased importance of obesity for T2D prevalence in these counties. Obesity ranked number one and the second most important variable in 60% and 25% of counties, respectively (Table 3). Physical inactivity was the 1st most influential risk factor in 30% of counties. These counties are distributed in Midwest counties and, to some extent, in the Northeast (Fig. 6g). Poverty demonstrated sparse local feature importance in the West and Midwest (Fig. 6k) and ranked first and second in 6.7% and 28% of counties, respectively.

**Table 3:** The proportion of counties with local risk factors (the risk factor with the 1st, 2nd, and 3rd highest value of local variable importance) on the county-level T2D prevalence.

Risk factors	Proportion of counties		
	1st	2nd	3rd
Obesity	60.6	24.5	6.0
Physical inactivity	29.9	38.4	19.4
Access to exercise	0.0	1.6	4.6
Food environment index	0.0	1.1	16.0
Poverty	6.6	28.0	32.4
Education	0.7	4.3	19.3

Only in < 1 county was education the most important risk factor for T2D. However, in only a few counties, access to exercise and food environment index ranked second and third (Table 3) in their importance as risk factors for county-level T2D prevalence. Generally, however, features with little importance to county-level T2D prevalence include access to exercise (Fig. 6h), food environment index (Fig. 6i), and education (Fig. 6l). Poverty demonstrated sparse local feature importance in the West and Midwest (Fig. 6k). Spatial variation of the local Gini index or IncNodePurity of the risk factors (Figure S7b–g) are similar to that of incMSE (Fig. 6f–k). There is slight variation in the spatial pattern in incMSE between 5 year (2013–2017) mean data (Fig. 6f–k), 3 year (2013–2015) mean data (Figure S8b–g), and for 2016 (Figure S8b–g) and 2017 (Figure S9b–g).

### Predictive performance of GW models

The performance of GW-RF and GW-OLS was evaluated using tenfold cross-validation. We found that the GW-RF model performed better than GW-OLS. The RMSE in GW-RF in cross-validation tests was 0.96% and explained 96% of the variability of T2D prevalence (Figure S11d). The scatter plots show that T2D prevalence predicted by GW-RF (Figure S11d) are closer to the 1:1 line than GW-OLS model (Figure S11c). However, residuals show the difference between the local trends and are less smooth, and a large number of counties showed positive residuals (Figure S11d).

**Figure 7:**
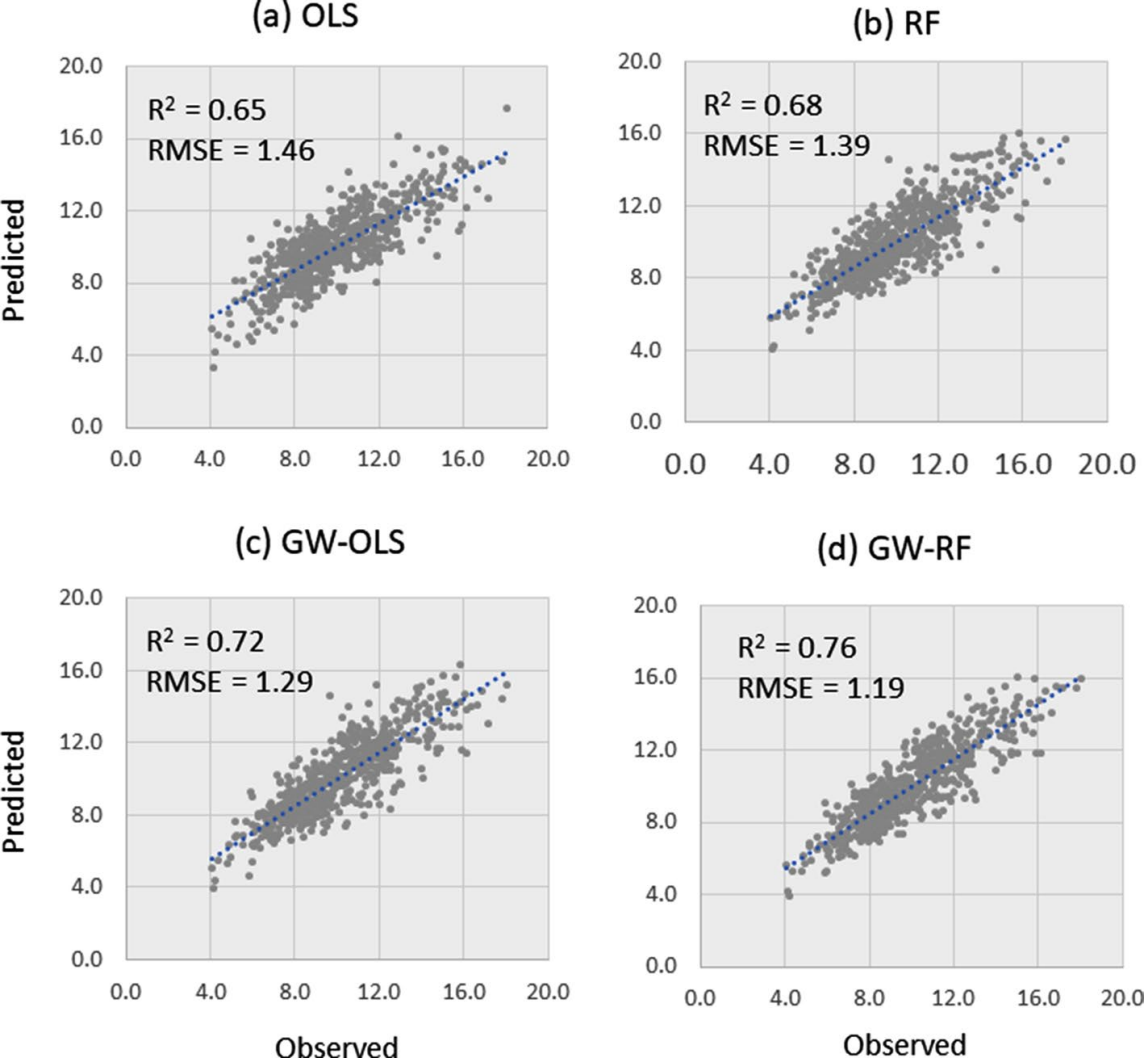
1:1 plot of observed versus predicted T2D prevalence (%) in 624 test counties for the (**a**) OLS, (**b**) RF, (**c**) GW-OLS, and (**d**) GW-RF regression models. All models were trained with data from 2484 counties (see supplementary information and Figure S2).

We further evaluated the performance of GWR with a subset test data set. The data set (n = 3108) was randomly split into 2484 training data used to train the GW models and 624 test data (Figure S2), which were used for evaluating the model performance. Figure 7 shows 1:1 plot that compares the observed to the predicted T2D prevalence using (a) OLS, (b) RF, (c) GW-OLS, and (d) GW-RF models. The plots show consistent improvements in accounting for T2D variability (R^2^) and RMSE when moving from the OLS to RF global models and then improve even further when comparing the GW-RF to the GW-OLS. The GW-RF model accounts for slightly more variability in T2D (R^2^ = 0.76) than the GW-OLS model (R^2^ = 0.72) with a concomitant reduction in RMSE values (1.19 vs 1.29).

## Discussion

This study presents a non-parametric geographically weighted model, the GW-RF model, as both a predictive and exploratory tool to describe spatial heterogeneity of association between T2D prevalence and six risk factors across United States counties. The GW-RF model with obesity, physical inactivity, access to exercise, food environment index, poverty, and education level inputs explained higher variability of the T2D prevalence than the traditional global (OLS and RF) and local (GW-OLS) models. GW-OLS has been widely used to explore the association between T2D and demographic, social, and environmental risk factors^10,18–20^. However, in most cases, GW-OLS does not consider the relationships among the predictors (collinearity), which is spatially heterogeneous in its correlation structure^37^. Spatial collinearity leads to parameter redundancies, which invalidate any attempt to interpret a single GW coefficient independent of the remaining local estimates at the same location^21^ and may affect the prediction performance of a GW regression model^37^. The GW-OLS, moreover, overlooks possible dependencies among the local regression coefficients associated with diverse exogenous variables^21^.

On the other hand, there is little or no agreement of collinearity in interpreting the feature importance in RF^46,47^. Collinearity influences variable importance in large-scale learning problems with dimensional data^48^. A review of various methods with highly correlated simulated data showed that RF was among the best performing models (lowest RMSE) in its ability to predict a training data set^49^ and can handle collinearity better than other models, though we do caution that this method may not be the most optimal in dealing with multicollinearity^50^. When some of the variables are not relevant for predicting the outcome of interest, a small perturbation of the training sample may completely change the ranking of the variables^51^. A model with a small number of predictors is more interpretable and improved model accuracy, as we observed in this study. However, it has been shown that feature selection based on built-in method that calculates feature importance based on incMSE is less suitable for data with multicollinearity^52^. Permutation based feature importance in correlated data may decrease the importance of the correlated features by splitting the importance between features.

Also, sensitivity to outliers is one of the critical drawbacks of the linear model, and a strong assumption is required. Similar to global RF, GW-RF has no linearity assumption since the tree-based model does not use metric distances between data points but applies splits along a tree^53^. Moreover, the GW-RF model showed higher predictive power when compared to GW-OLS and global RF models. The application of GW-RF would produce the enhanced generalizability of the data created by the RF model in addition to the consideration of spatial heterogeneity that occurs by accounting for the localities present in the spatial data.

Several spatial modeling approaches have demonstrated an association between county-level diabetes prevalence and obesity^8–10^. In this study, GW-RF ranked obesity as the most important risk factor in many counties (30–60%). These counties are mostly in the West and Northeast (low-low cluster) and South (high-high cluster) regions. In general, the 5 year mean of T2D prevalence in the South region of the United States is higher than that of the national average. This region of the US, where rates of diabetes tend to be historically high, has been termed the "diabetes belt," referring to the continuity of counties that are and are adjacent to high diabetes prevalence areas^8^. The high T2D prevalence can be explained by a higher prevalence of obesity, poverty, and low populations of the Mississippi valley^8^ as well as those in the Appalachian regions^54^. Further, diverse populations living in impoverished and poorly integrated communities have similar diabetes prevalence^55,56^. Many counties that have high diabetes prevalence are outside of the diabetes belt. For example, some counties in North Dakota, South Dakota, Oklahoma, New Mexico, and Nebraska had a prevalence of diabetes > 15% but are not included in the belt. Many of these counties are characterized by extreme poverty, and some have large Native American populations with a relatively high prevalence of diabetes^57^.

The built environment or community characteristics are a strong determinant of an individual's physical activity, diet, and risk of obesity^58^ and T2D^59,60^. We found the food environment index, which measures food access and availability^33^ was negatively related to T2D prevalence. However, the local relationship between T2D and food environment index was weaker than that of obesity, physical inactivity, and poverty. Only in few counties, it ranked as 2nd or 3rd most important variables. Food-insecure populations are likely to have limited access to healthy food^61^ and usually depend on more convenient, high calorie foods, which can contribute to obesity and increased risk of T2D^62,63^. Areas with low income and low physical access to food^64^ are correlated with a high prevalence of obesity as supermarkets traditionally provide healthier options than convenience stores or smaller grocery stores^65^. The components of the health and food environment were found to be associated with T2D prevalence^11–13, 66^. However, the results of these studies are mostly inconsistent and unexpected in terms of associations shared with T2D prevalence. For instance, fast food restaurants serving high calorie foods, typically to low socioeconomic status communities, were negatively correlated with T2D prevalence in counties of South Carolina^11^. Another study utilizing OLS found that in the diabetes belt, fast food restaurant density was a positive predictor of T2D prevalence. In contrast, in the remainder of the United States, the associate was negative^6^. Geodemography techniques applied to the diabetes belt identifying correlates of diabetes at finer, tapestry scales^67^ also challenge some findings that T2D prevalence is high in areas with high minority compositions and urban living^10^. A longitudinal analysis recently showed that food insecurity was associated with higher HbA1c, but living in an area with low physical food access was not^64^. The inconsistencies in these findings may be due to the varying strength of relationships between these environmental and social variables and T2D, which are poorly captured by linear models.

Besides food environments, living in closer proximity to sidewalks, parks, and gyms are more likely to have access to exercise and physical activity^68–70^. Physical inactivity is not solely associated with community characteristics^71^. Physical inactivity has been associated with T2D prevalence, independent of obesity^72^ and related to high health care expenditures^73^. In general, individuals residing in counties with high rates of poverty tend to live in environments with limited access to safe sidewalks, parks, and gyms.

The T2D prevalence data used in this study are CDC county-level estimates which have been used in many studies^1,8,9^. However, these data have several limitations. The county-level prevalence data are model-based estimates from the BRFSS telephone survey, which has some inherent limitations (e.g., recall bias, social desirability bias, inability to reach houses without landline telephones prior to 2011)^8^. Diabetes prevalence excludes persons with undiagnosed diabetes^9^, which might affect the results if counties significantly varied in the proportion of undiagnosed diabetes^8^. Underestimated body weight and overestimated height by self-report have historically underestimated county-level obesity prevalence^74^.

Besides the limitation related to the data, GW models themselves have several limitations. In any GW model, local regression coefficients or local variable importance are derived in locations (eg, counties) based on the most proximate area of interest. Unlike the global model, the GW model is calibrated locally rather than globally; at each location or county in our study, a GW model was fitted, considering only nearby county data. We used adaptive kernel bandwidth to select the optimum number of counties to train GW models accounting for the differences in the size of the county and, therefore, the distance of influence, which is theoretically unknown and perhaps inconsistent across a geographic area^10^. Because of this inconsistency, the number of neighbors or bandwidth were estimated based on the characteristics of proximate counties as defined by the kernel type, which may lead to spillover effects of the dependent variable in neighboring counties or the residuals' spatial autocorrelation. However, it is a typical problem in the spatial modeling of infectious disease^75^.

There are also limitations to our findings. The local R^2^ in GW-OLS and GW-RF model with six risk factors are more robust (pseudo-R^2^ > 0.7) in many counties. At the same time, they become less accurate (pseudo-R^2^ < 0.5) in the central Midwest and southern Texas. The result suggests that additional variables should be included to improve the performance of the GW model further in these regions.

Although the GW-RF model in this study used only six well-known risk factors for exploring spatial heterogeneity of T2D prevalence, the focus of this study is not understanding the causation of T2D prevalence across US counties. Instead, this study is intended as a demonstration of how the recently developed GW-RF model^23,24,76,77^ can be used as both a predictive and exploratory tool to explore spatial heterogeneity of T2D considering the non-linear relationship between risk factors and T2D prevalence. Thus, this method is applicable in many instances where there is an issue about selecting significantly correlated variables at various geographical locations.

## Conclusions

This study is the first to our knowledge to apply the GW-RF regression model to explore spatial heterogeneity of county-level T2D prevalence in relation to multiple risk factors. We demonstrated improved goodness-of-fit and enhanced predictability by a GW-RF model against traditional local and global models. One of the important contributions of this study is the ranking of US counties according to six major risk factors associated with T2D prevalence. Although there is a clear consistency between GW-OLS and GW-RF model for predicting T2D prevalence, it is evident that GW-RF performed better than GW-OLS model. The GW-RF may be applicable in spatial models where multicollinearity at various geographical locations is a major concern.

The results of this study may also present opportunities for focused epidemiologic research at the county level to better understand the mechanisms driving T2D prevalence in various regions. The findings of this study may lead to more tailored and effective prevention strategies from a policy perspective, which is critical, given the projected prevalence increase of diabetes in the coming decades. Understanding the spatial heterogeneity of the associations between T2D and risk factors may enable more advanced research and policy development to address the underlying, spatially varying contributors to T2D across US counties.

## Supplementary Information

Supplementary Information.

## Data Availability

The data sets generated during this study are available from the corresponding author upon reasonable request.
