# Author Correction: Geographically weighted machine learning model for untangling spatial heterogeneity of type 2 diabetes mellitus (T2D) prevalence in the USA

**DOI:** 10.1038/s41598-021-97279-3

**Published:** 2021-08-30

**Authors:** Sarah Quiñones, Aditya Goyal, Zia U. Ahmed

**Affiliations:** 1 University at Buffalo, State University at New York, Buffalo, USA; 2 Research and Education in Energy, Environment, and Water (RENEW) Institute, University at Buffalo, State University at New York, 108 Cooke Hall, Buffalo, NY 14260 USA

Correction to: *Scientific Reports* 10.1038/s41598-021-85381-5, published online 26 March 2021

The original version of this Article contained an error in the Materials and methods section, under the subheading ‘Data’, where

“Estimates of county-level prevalence were age-adjusted using the 2000 United States standard population using the following age groups: 20–44, 45–64, and 65 and older^28^.”

now reads:

“Estimates of county-level prevalence were age-adjusted using the 2000 United States standard population using the following age groups: 20–44, 45–64, and 65 and older^28^. Since T2D accounts for 90–95% of all types of diabetes, we have used T2M to represent USDSS county-level diabetes prevalence.”

In addition, in the Discussion section,

“Several spatial modeling approaches have demonstrated an association between county-level T2D prevalence and obesity^8–10^.”

now reads:

“Several spatial modeling approaches have demonstrated an association between county-level diabetes prevalence and obesity^8–10^.”

The original Article has been corrected.

