# Supplementary material for: Geographically weighted machine learning model for untangling spatial heterogeneity of type 2 diabetes mellitus (T2D) prevalence in the USA
Source: Sci Rep. 2021 Mar 26;11:6955. doi: 10.1038/s41598-021-85381-5 (PMC7997882; doi:10.1038/s41598-021-85381-5)
Supplement: Supplementary file 1 — Supplementary Information. [file 41598_2021_85381_MOESM1_ESM.docx]

**Supplementary Information**

**Geographically Weighted Machine Learning Model for Untangling Spatial Heterogeneity of Type 2 diabetes mellitus (T2D) Prevalence in the USA**

**Sarah Quinones^1^, Aditya Goyal^2^ and Zia U. Ahmed^2^***

**^1^Sarah Quinones**

University at Buffalo, State University at New York, United States

Email: [squinone@buffalo.edu](mailto:squinone@buffalo.edu)

**^2^Aditya Goyal**

Research and Education in Energy, Environment, and Water (RENEW) Institute

University at Buffalo, State University at New York, United States

Email: [adigoyal101@gmail.com](mailto:adigoyal101@gmail.com)

**^2^Zia U. Ahmed***

University at Buffalo, State University at New York, United States

Research and Education in Energy, Environment, and Water (RENEW) Institute

108 Cooke Hall, Buffalo, NY 14260
Phone: 716-645-1405
Email: [zahmed2@buffalo.edu](mailto:zahmed2@buffalo.edu)

*** Corresponding author**

## Hot Spot Analysis (Getis-Ord Gi*)

## The Hot Spot Analysis calculates the Getis-Ord Gi* statistic (pronounced G-i-star) for each feature in a dataset ^1^. The resultant -Ord Gi* statistics (*z*-score and *p*-values) indicate areas with either high or low-value clusters. Larger z-scores (statistically significant positive z-scores) show more intense clustering of high values, and smaller z-score (statistically significant negative *z*-scores), suggest more intense clustering of low values.

The Getis-Ord local statistic is given as:

$G_{i}^{*}= \frac{\sum_{j=1}^{n} w_{i,j}x_{j}- \bar{X}\sum_{j=1}^{n} w_{i,j}}{S\sqrt{\frac{[n\sum_{j=1}^{n} w_{i,j}^{2}-{(\sum_{j=1}^{n} w_{i,j})}^{2}]}{n-1}}}$ [1]

where *x_j_* is the attribute value for feature *j*, *w_i,j_* is the spatial weight between feature *i* and *j*, *n* is equal to the total number of features.

Getis-Ord Gi* Hot Spot map was created in ArcGIS Desktop 10.6.1^2^

**Bivariate Moran-I**

Bivariate Moran-I describes the relationship between the value for one variable at location *i*, *xi*, and the average of the neighboring values for another variable, i.e., its spatial lag ∑*jwijyj*. This statistic is the product of *xi* with the spatial lag of *yi* (i.e., ∑*jwijyj*), with both variables standardized, such that their means are zero and variances equal one:


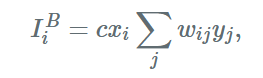
 **[2]**

where *wij* are the elements of the spatial weights matrix.

We used GeoDa (1.14) for Bivariate Moran-I analysis ^3^

**Geographically Weighted Regression (GWR)**

Geographically weighted regression (GWR) is a spatial analysis technique mainly intended to indicate where non-stationarity is taking place on the space allowing the relationships between the independent and dependent variables to vary by locality. Limitations of GWR include problems of multicollinearity and the approaches to calculating goodness of fit statistics. GWR is a modification to classical regression modeling, namely the ordinary least squares (OLS) regression ^4^. The basic form of the GW regression model is:

$y_{i}=\beta_{i0}+ \sum_{k=1}^{m} \beta_{ik}x_{ik}+ \epsilon_{i}$ [3]

where *y_i_* is the dependent variable at location *i*; *X*_ik_ is the value of the *kth* independent variable at location *i*; *m* is the number of independent variables; *β_i0_* is the intercept parameter at location *i*; *β_ik_* is the local regression coefficient for the *kth* independent variable at location *i*; and ɛ_i_ is the random error at location *i*.

**Bi-square kernels for Bandwith selection**

Geographical weighting solely applies to the data in all GW methods, where each local model is fitted to its own GW data (sub-) set. The size of the window over which this localized model might apply is controlled by the kernel function’s bandwidth ^5^. A bandwidth can be specified either as a fixed (constant) distance or as a fixed (constant) number of local data (i.e. an adaptive distance). For a continuous kernel function, a bandwidth can be specified either as a fixed distance or as a 'fixed quantity that reflects local sample size' (i.e. still an 'adaptive' distance but the actual local sample size will be the sample size as functions are continuous). In practise a fixed bandwidth suits fairly regular sample configurations whilst an adaptive bandwidth suits highly irregular sample configurations. Adaptive bandwidths ensure sufficient (and constant) local information for each local calibration. The bi-squre weight kernel define as:


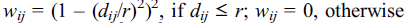
 **[4]**

where j is the index of the observation point and dij the distance between the points indexed by i and j. The bandwidth r can be specified beforehand (i.e. a fixed distance) or specified as the distance between

the point i and its Nth nearest neighbor, where N is specified beforehand (i.e. an adaptive distance). The bi-square function gives fractional decaying weights according to the proximity of the data to each point i, up until a fixed distance or a distance according to a specified Nth nearest neighbor.

**Random Forest (RF)**

The Random Forests method uses an ensemble of multiple iterations of decision trees where each tree is made by bootstrapping of the original data set. It allows for robust error estimation with the remaining test set, the so-called Out-Of-Bag (OOB) sample. The excluded OOB samples are predicted from the bootstrap samples and by combining the OOB predictions from all trees. The RF algorithm can outperform linear regression ^6^, and unlike linear regression, RF has no requirements concerning the form of the probability density function of the target variable ^7^. RF allows estimation of the importance of variables as measured by the mean decrease in prediction accuracy before and after permuting OOB variables. The difference between the two is then averaged over all trees and normalized by the standard deviation of the differences.

The algorithm flow of the RF is as follows ^8^:

1. The *n* data sets *D*_1_, *D*_2_, ⋯, *D_n_* are extracted by repeatedly using the bootstrap method to randomly extract the whole dataset *D*, and the corresponding *n* decision trees *H*_1_, *H*_2_, ⋯, *H_n_* are generated.
2. At each node of the decision tree, randomly select *m* (*m* < *k*) variables from all the *k* variables of the decision tree, and each node is split using the selected *m* variables by the optimal segmentation method determined by a segmentation criterion.
3. The value of *m* remains unchanged while the forest grows. Each tree grows to its largest extent without pruning until it cannot be split. Thus, the correlation between the decision trees in the forest decreases through a random selection of variables at each node of the tree and the optimal split of each node is determined by the selected variables only, instead of all variables. Each tree can grow to its most considerable extent without pruning. Therefore, the algorithm can deal with excessive redundant features and avoid overfitting.

**Geographically Weighted Random Forest (GW-RF)**

Geographically Weighted Random Forest (GW-RF) is an extension of the global consisting of several local sub-models Georganos ^9^. The principle idea of GW-RF is similar to that of GWR ^4^ and inherits the merits of the RF, making the RF from being applicable from a global system to a local system by integrating spatial weight matrix (SWM) and RF into a local regression analysis framework ^8^. Thus, it can handle high-dimensional variables with nonlinear relationships and multicollinearity. The variable importance for each spatial unit can be obtained from the GW-RF.

The GW-RF can be explained by extending the following linear regression equation (5):

$Y_{i}= {ax}_{i}+e,i=1:n$ [5]

where *Yi* is the value of the dependent variable for the ith observation and *axi* is the nonlinear prediction of RF-based on a set of *x* independent variables, with e being an error term

The SWM for each spatial unit of the study area should first be made according to the specified spatial weight rule. The SWM for the whole study area with *p* spatial units can be expressed as in Eq. [5]:


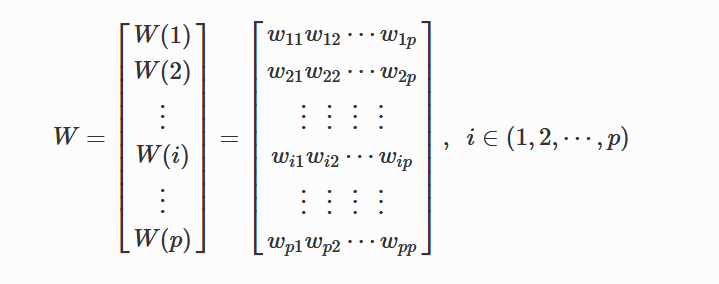
 [6]

The GW-RF equation,

$Y_{i}=a\left( u_{i}, v_{i} \right)x_{i}+e,i=1:n$ [7]

In equation 6, a(ui,vi)x is the prediction of an RF model calibrated on location *i*, and (*ui,vi*) are the coordinates.

**Root-mean-square error** (**RMSE**)

The  root-mean-square error (RMSE) is a frequently used measure of the differences between values (sample or population values) predicted by a model or an [estimator](https://en.wikipedia.org/wiki/Estimator) and the values observed.

[8]

[1]

Where, *n* is the number of counties, $y$ and $\hat{y}$are observed and predicted LBC rates in county *i*.

Table S1. Summary statistics of mean type-2 diabetes and six risk factors of all, train and test counties.

|  | All Counties | | | | | | | |
| --- | --- | --- | --- | --- | --- | --- | --- | --- |
|  | N | Mean | Median | Min. | Max | Q25 | Q75 | SD |
| Diabetes | 3108 | 10.0 | 9.7 | 3.5 | 20.9 | 8.2 | 11.6 | 2.5 |
| Obesity | 3108 | 32.1 | 32.5 | 8.5 | 48.9 | 29.2 | 35.1 | 4.7 |
| Physical Inactivity | 3108 | 25.3 | 25.2 | 4.5 | 40.2 | 22.1 | 28.5 | 4.8 |
| Access Excercise | 3108 | 59.8 | 61.6 | 0.0 | 100.0 | 45.6 | 75.6 | 21.7 |
| Food Envrionment Index | 3108 | 7.3 | 7.5 | 1.3 | 10.0 | 6.7 | 8.0 | 1.2 |
| Education | 3108 | 44.2 | 44.3 | 11.6 | 86.8 | 35.9 | 52.4 | 11.4 |
| Poverty | 3108 | 16.3 | 15.4 | 3.3 | 53.7 | 11.7 | 19.6 | 6.3 |
|  |  |  |  |  |  |  |  |  |
|  | Training | | | | | | | |
| Diabetes | 2484 | 10.0 | 9.7 | 3.5 | 20.9 | 8.1 | 11.6 | 2.5 |
| Obesity | 2484 | 32.1 | 32.4 | 8.5 | 48.9 | 29.2 | 35.1 | 4.7 |
| Physical Inactivity | 2484 | 25.3 | 25.3 | 4.5 | 40.2 | 22.1 | 28.5 | 4.9 |
| Access Excercise | 2484 | 59.6 | 61.2 | 0.0 | 100.0 | 45.4 | 75.0 | 21.8 |
| Food Envrionment Index | 2484 | 7.3 | 7.5 | 1.3 | 10.0 | 6.7 | 8.0 | 1.2 |
| Education | 2484 | 44.3 | 44.3 | 12.3 | 86.8 | 35.9 | 52.5 | 11.6 |
| Poverty | 2484 | 16.3 | 15.4 | 3.3 | 53.7 | 11.6 | 19.6 | 6.3 |
|  |  |  |  |  |  |  |  |  |
|  | Test | | | | | | | |
| Diabetes | 624 | 10.0 | 9.7 | 4.1 | 18.1 | 8.3 | 11.5 | 2.4 |
| Obesity | 624 | 32.0 | 32.5 | 14.3 | 45.8 | 29.1 | 35.0 | 4.6 |
| Physical Inactivity | 624 | 25.2 | 25.1 | 9.6 | 39.2 | 22.2 | 28.4 | 4.7 |
| Access Excercise | 624 | 60.9 | 62.4 | 1.0 | 100.0 | 46.4 | 77.2 | 21.3 |
| Food Envrionment Index | 624 | 7.3 | 7.5 | 1.4 | 9.3 | 6.8 | 8.0 | 1.1 |
| Education | 624 | 44.2 | 44.8 | 11.6 | 71.4 | 36.0 | 51.9 | 10.7 |
| Poverty | 624 | 16.2 | 15.3 | 4.6 | 44.1 | 12.2 | 19.3 | 6.2 |

Table S2. List of hyper-parameters used to conduct the grid-search and to select the best parameters for the random forest (RF) regression model.

| **max_depth** | **min_rows** | **mtries** | **ntrees** | **sample_rate** | **rmse** |
| --- | --- | --- | --- | --- | --- |
|  |  |  |  |  |  |
| 50 | 2 | 4 | 2,950 | 0.5 | 1.363 |
| 10 | 5 | 4 | 3,300 | 0.8 | 1.3648 |
| 20 | 5 | 4 | 550 | 0.8 | 1.3678 |
| 50 | 1 | 4 | 2,684 | 0.6 | 1.3739 |
| 20 | 5 | 5 | 1,350 | 0.8 | 1.3771 |
| 50 | 2 | 5 | 4,500 | 0.7 | 1.3818 |
| 30 | 5 | 5 | 2,750 | 1 | 1.4115 |

**Figure S1**. County-level diabetes prevalence and six factors. Maps were created in the R (version 4.0.0) Statistical Computing Environment ^10^.


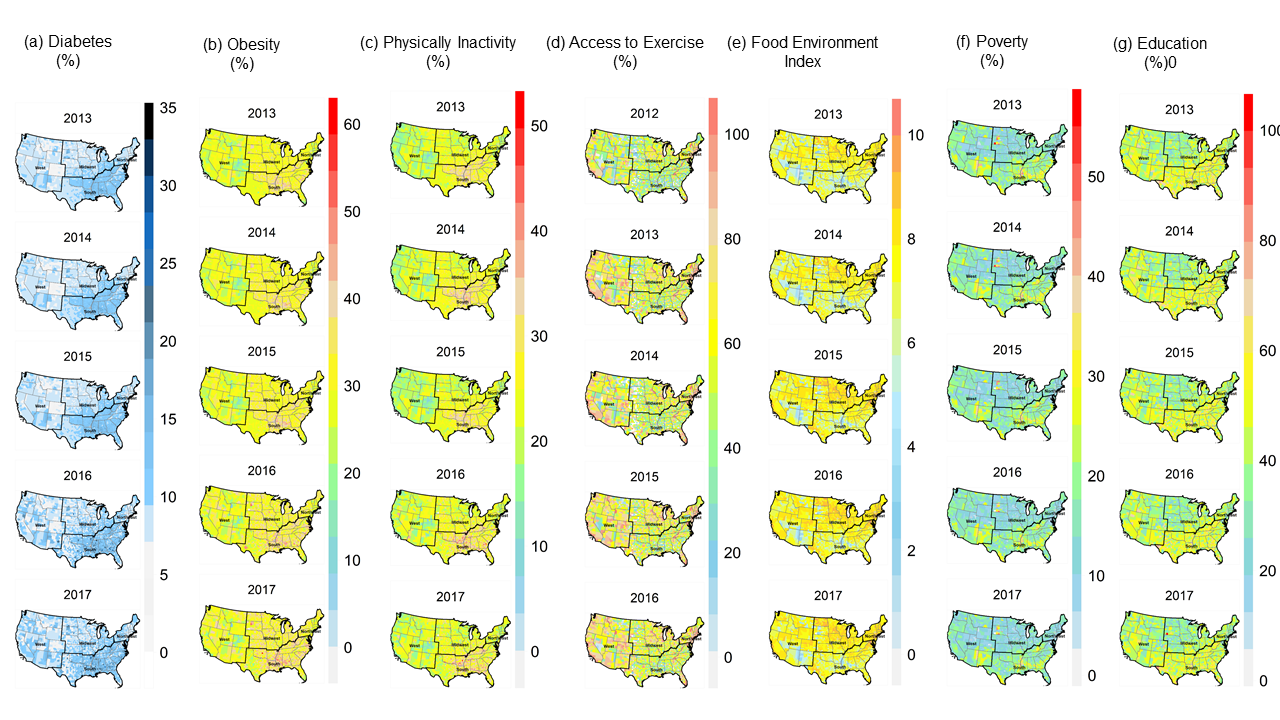


**Figure S2**. Spatial variability of mean diabetes prevalence in training and test counties . Maps were created in the R (version 4.0.0) Statistical Computing Environment ^10^.


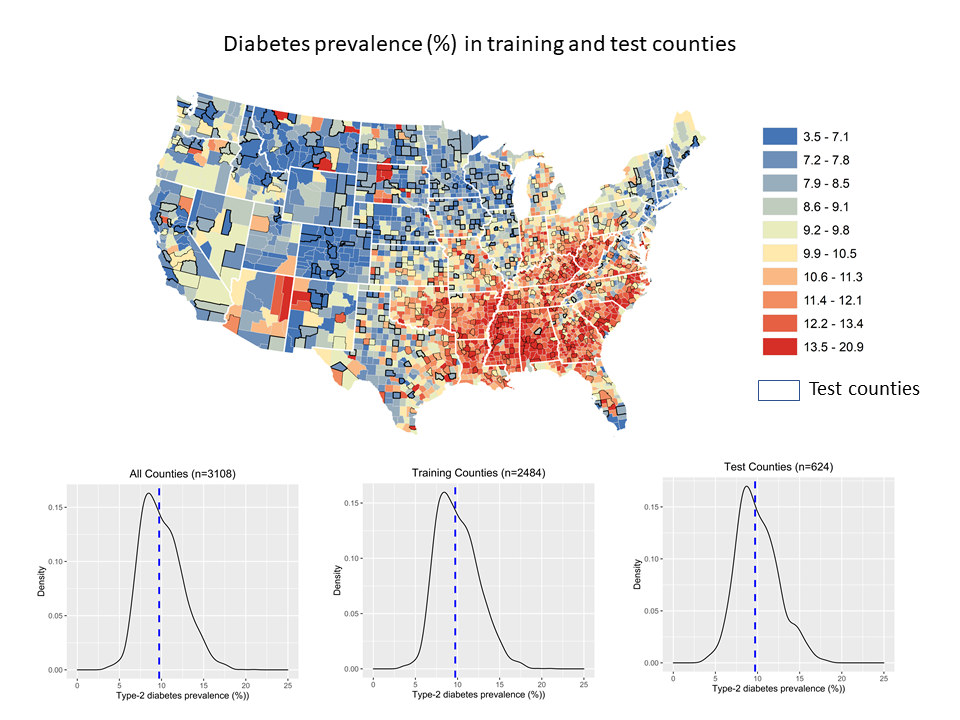


**Figure S3.** AICc values in relation to number of neigbors


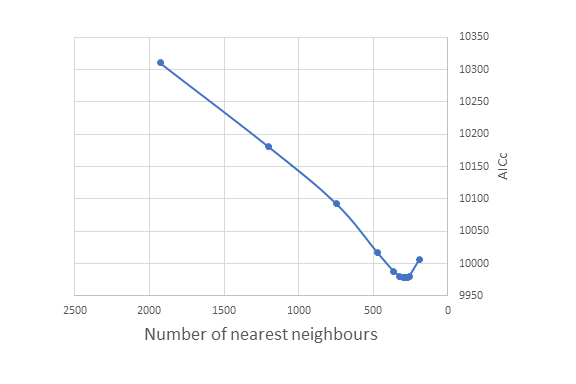


**Figure S4**. Global bivariate Moran’s I


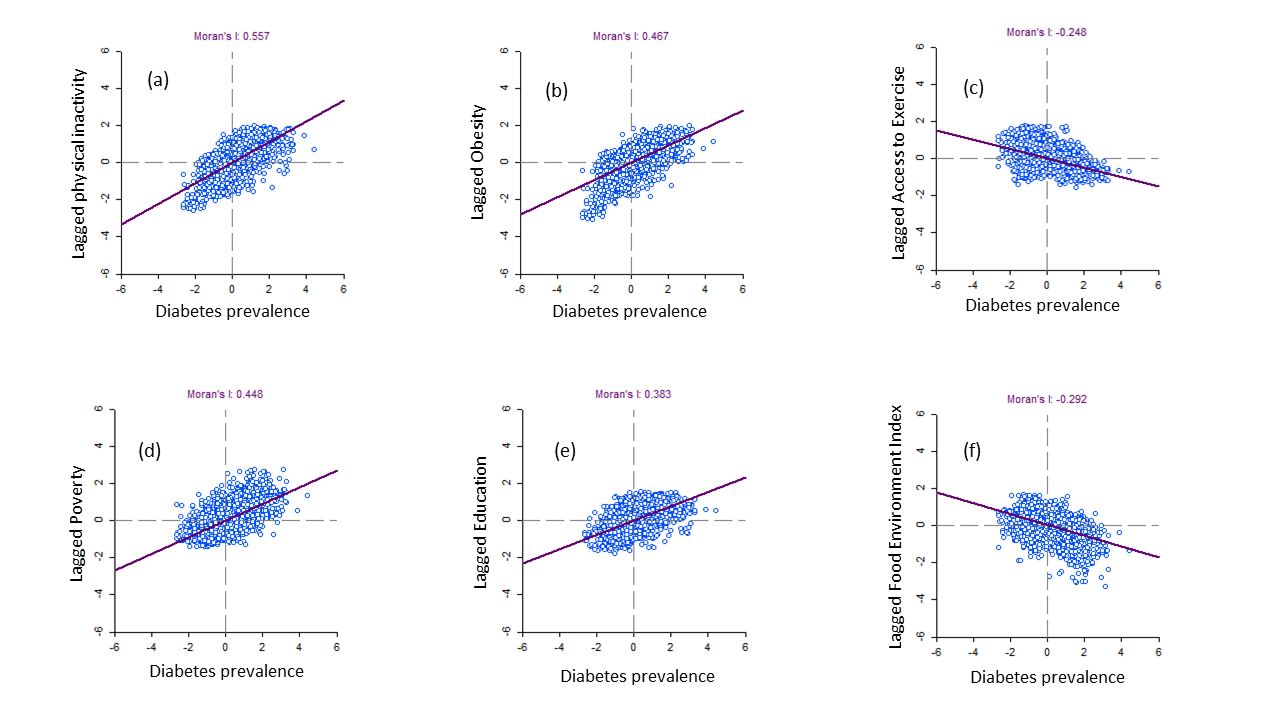


**Figure S5**. Local variance inflation factors (VIFs) of (a) obesity, (b) physical inactivity, (c) access to exercise, (d) food environment index, (e) poverty, and (f) education. Maps were created in the R (version 4.0.0) Statistical Computing Environment ^10^.


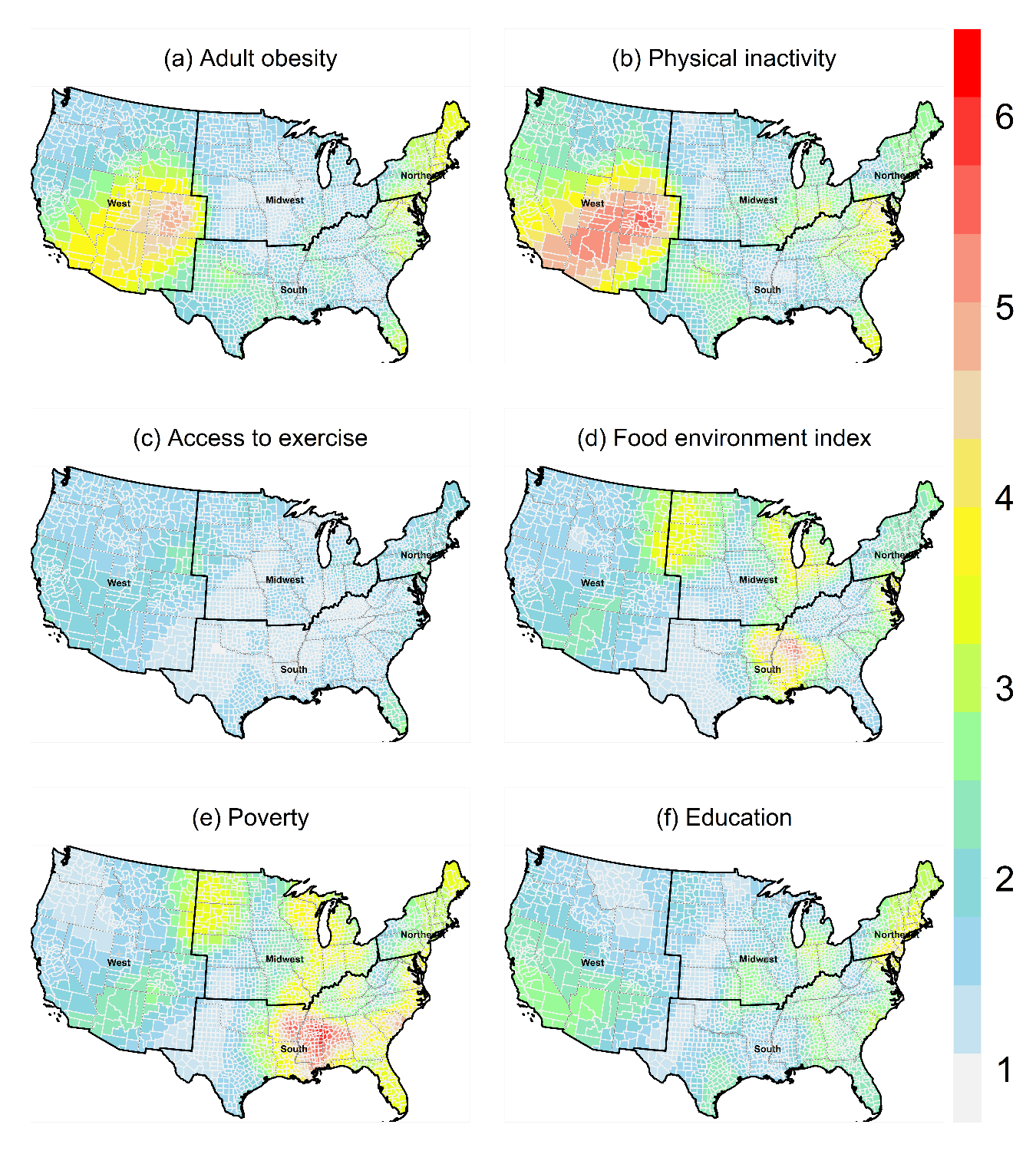


**Figure S6**. Maps show the counties where local coefficients of geographically weighted OLS (GR-OLS) values are positive (obesity, physical inactivity, poverty, education) and negative (access to exercise and food environment index), and adjusted *p*-values are significant (< 0.05). Maps were created in the R (version 4.0.0) Statistical Computing Environment ^10^.


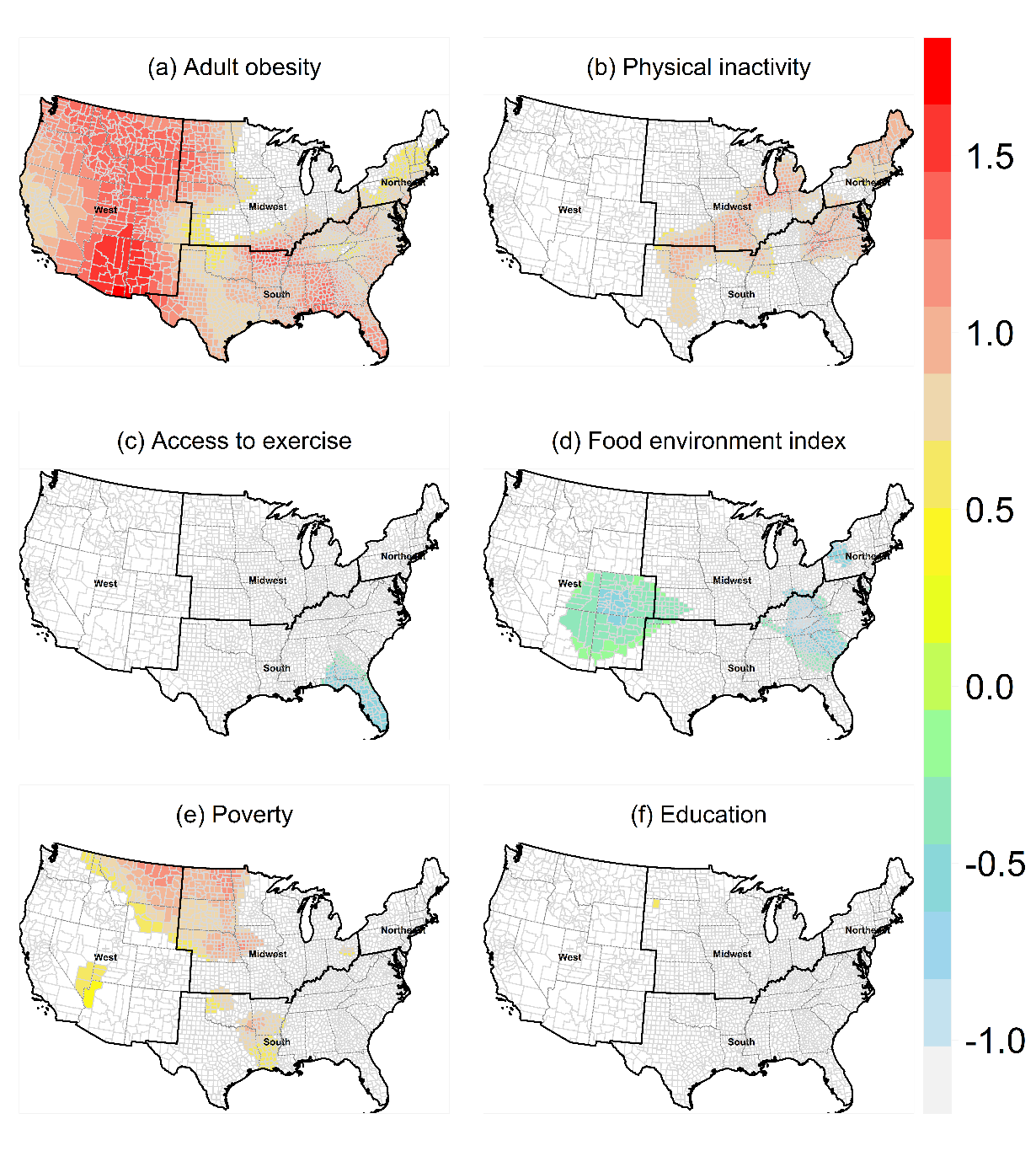


**Figure S7.** (a) Mean Decrease Gini impurity index (IncNodePurity) of global random forest model, and (b-g) spatial variation of mean Gini decrease impurity index (IncNodePurity)of obesity, physical inactivity, access to exercise, food environment index, poverty, and education in geographically weighted random forest regression models. Higher values imply increased importance. The random forest model was trained with five years of mean data (2013-2017) of 3108 counties. Maps were created in the R (version 4.0.0) Statistical Computing Environment ^10^.


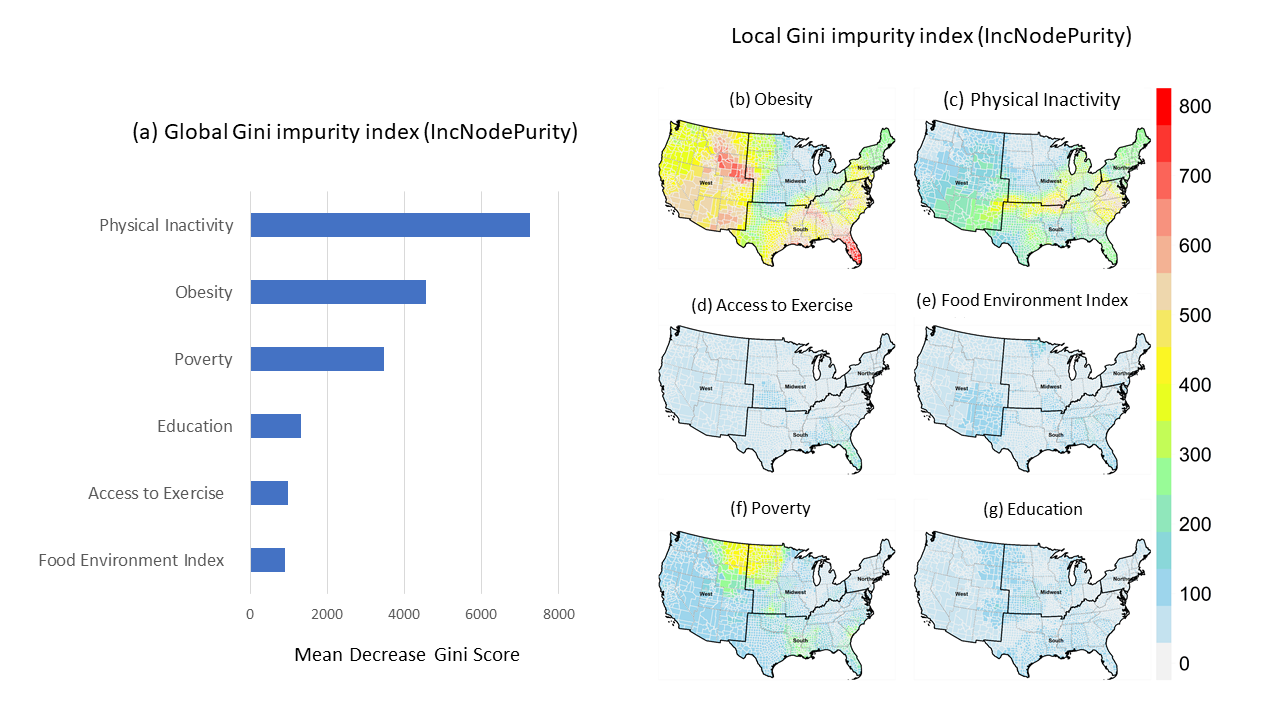


**Figure S8.** (a) Permutation-based feature importance from global random forest, (b-g) spatial variation of local feature importance (incMSE) of obesity, physical inactivity, access to exercise, food environment index, poverty, and education in geographically weighted random forest regression models. Higher values imply increased importance. The random forest model was trained with of mean data from 2013 to 2015 of 3108 counties. Maps were created in the R (version 4.0.0) Statistical Computing Environment ^10^.

**
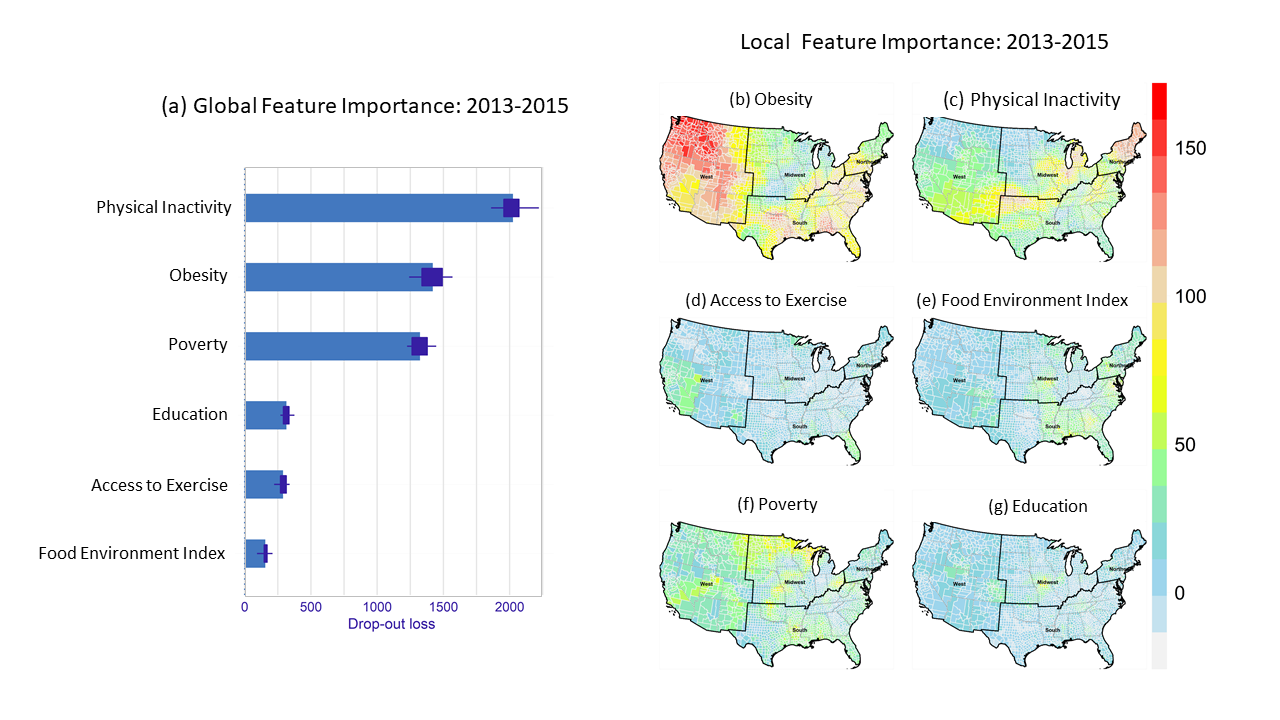
**

**Figure S9.** (a) Permutation-based feature importance from global random forest, (b-g) spatial variation of local feature importance (incMSE) of obesity, physical inactivity, access to exercise, food environment index, poverty, and education in geographically weighted random forest regression models. Higher values imply increased importance. The random forest model was trained with data for the year 2016 of 3108 counties. Maps were created in the R (version 4.0.0) Statistical Computing Environment ^10^.

**
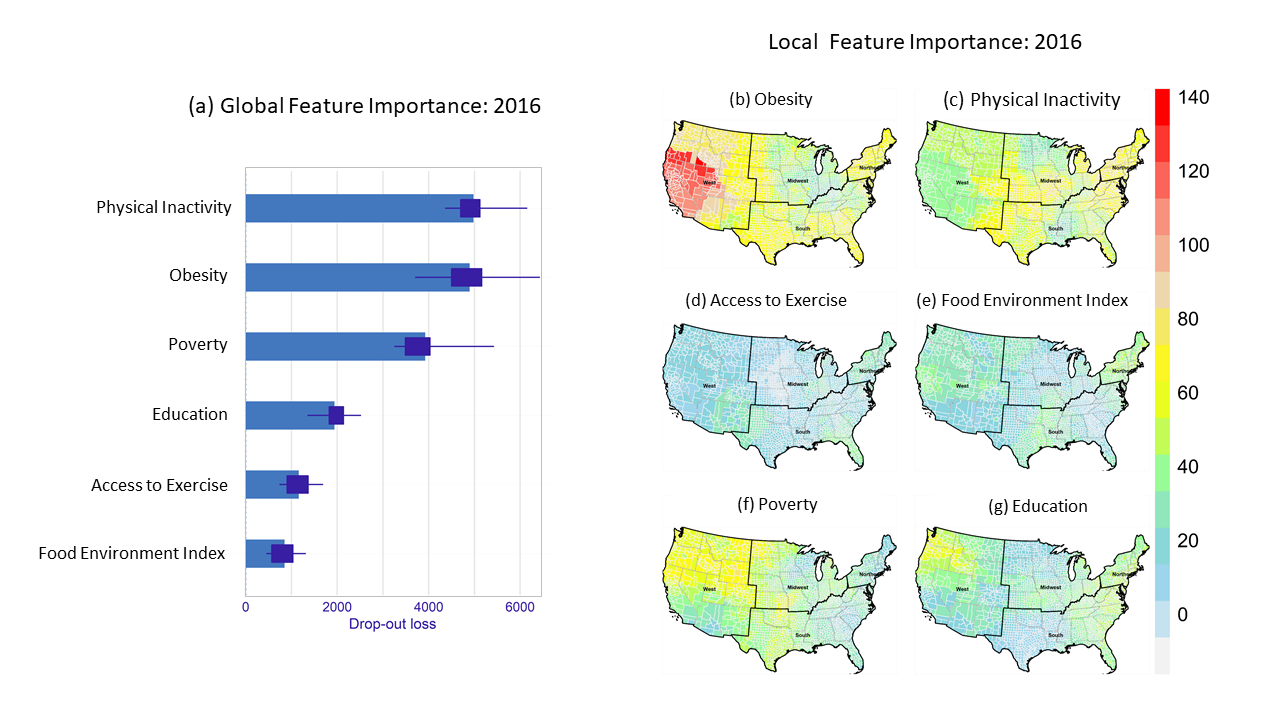
**

**Figure S10.** (a) Permutation-based feature importance from global random forest, (b-g) spatial variation of local feature importance (incMSE) of obesity, physical inactivity, access to exercise, food environment index, poverty, and education in geographically weighted random forest regression models. Higher values imply increased importance. The random forest model was trained with data for the year 2017 of 3108 counties. Maps were created in the R (version 4.0.0) Statistical Computing Environment ^10^.

**
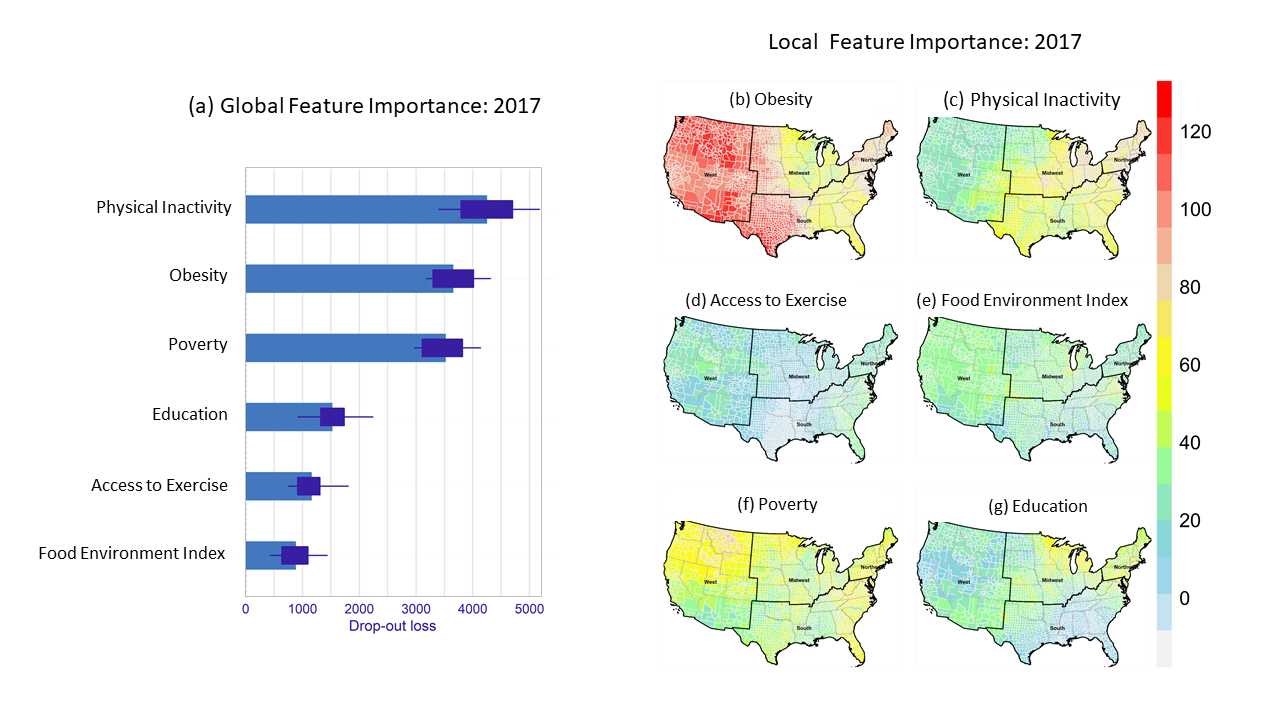
**

**Figure S11.** Local R^2^, 1:1 plots of 10-fold cross-validated observed vs predicted values and residuals of during training of local GWR and GR-RF models for the five years mean data (2013-2017). Maps were created in the R (version 4.0.0) Statistical Computing Environment ^10^.


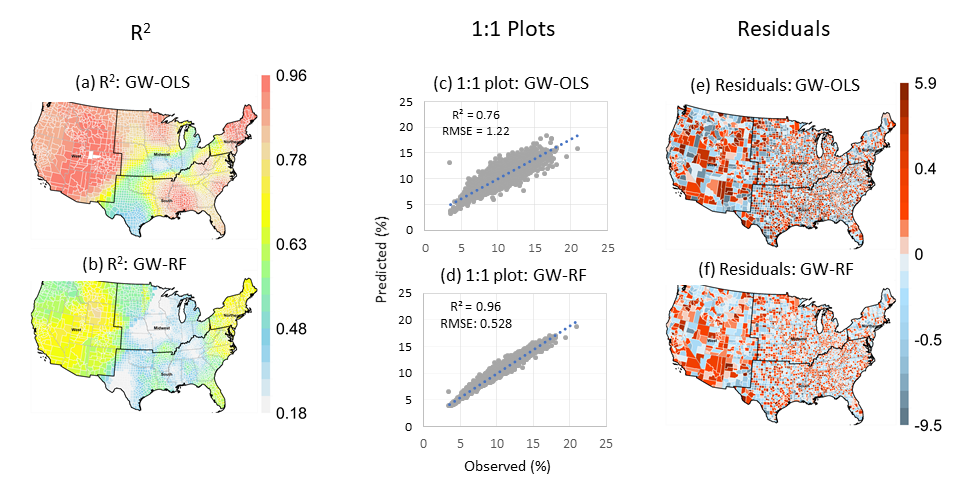


References:

1 Getis, A. & Ord, J. K. The Analysis of Spatial Association by Use of Distance Statistics. *Geographical Analysis* **24**, 189-206, doi:<https://doi.org/10.1111/j.1538-4632.1992.tb00261.x> (1992).

2 ESRI. ArcGIS desktop: release 10.6.1. *Environmental Systems Research Institute, CA* (2019).

3 Anselin, L., Syabri, I. & Kho, Y. in *Handbook of applied spatial analysis* 73-89 (Springer, 2010).

4 Fotheringham, A. S., Brunsdon, C. & Charlton, M. *Geographically weighted regression: the analysis of spatially varying relationships*. (John Wiley & Sons, 2003).

5 Lu, B., Harris, P., Charlton, M. & Brunsdon, C. The GWmodel R package: further topics for exploring spatial heterogeneity using geographically weighted models. *Geo-spatial Information Science* **17**, 85-101, doi:10.1080/10095020.2014.917453 (2014).

6 Hengl, T. *et al.* Mapping Soil Properties of Africa at 250 m Resolution: Random Forests Significantly Improve Current Predictions. *PLOS ONE* **10**, e0125814, doi:10.1371/journal.pone.0125814 (2015).

7 Kuhn, M. & Johnson, K. *Applied predictive modeling*. Vol. 26 (Springer, 2013).

8 Luo, Y., Yan, J. & McClure, S. Distribution of the environmental and socioeconomic risk factors on COVID-19 death rate across continental USA: a spatial nonlinear analysis. *Environmental Science and Pollution Research*, doi:10.1007/s11356-020-10962-2 (2020).

9 Georganos, S. *et al.* in *2019 Joint Urban Remote Sensing Event (JURSE).* 1-4.

10 R. R: A language and environment for statistical computing. R Foundation for Statistical Computing, Vienna, Austria. URL, <https://www.R-project.org/>. (2020).
